# Microscopic Evolution of Laboratory Volcanic Hybrid Earthquakes

**DOI:** 10.1038/srep40560

**Published:** 2017-01-11

**Authors:** H. O. Ghaffari, W. A. Griffith, P. M. Benson

**Affiliations:** 1 Department of Earth and Environmental Sciences, University of Texas at Arlington, Arlington, TX, 76019, USA; 2 Rock Mechanics Laboratory, School of Earth and Environmental Sciences, University of Portsmouth, Portsmouth, PO1 3QL, UK

## Abstract

Characterizing the interaction between fluids and microscopic defects is one of the long-standing challenges in understanding a broad range of cracking processes, in part because they are so difficult to study experimentally. We address this issue by reexamining records of emitted acoustic phonon events during rock mechanics experiments under wet and dry conditions. The frequency spectrum of these events provides direct information regarding the state of the system. Such events are typically subdivided into high frequency (HF) and low frequency (LF) events, whereas intermediate “Hybrid” events, have HF onsets followed by LF ringing. At a larger scale in volcanic terranes, hybrid events are used empirically to predict eruptions, but their ambiguous physical origin limits their diagnostic use. By studying acoustic phonon emissions from individual microcracking events we show that the onset of a secondary instability–related to the transition from HF to LF–occurs during the fast equilibration phase of the system, leading to sudden increase of fluid pressure in the process zone. As a result of this squeezing process, a secondary instability akin to the LF event occurs. This mechanism is consistent with observations of hybrid earthquakes.

Critical challenges remain in the study of dynamic interactions between *in-situ* liquids and moving defects (fractures and micro-defects such as dislocations and other topological defects) and in triggering the nucleation and/or movement of defects^1^,^2^,^3^. Such interactions might emit broadband phononic excitations which mirror the complexity of the source dynamics from which they are derived as well as the environment in which the sources propagate.

An important manifestation of these excitations in the geosciences is the study of the seismic response of rocks in the presence of pore fluids. Seismological observations of earthquakes associated with active volcanism have exposed a wide variety of physical phenomena that are manifested as seismic activity^4^,^5^. In particular, Low-Frequency (LF) earthquakes have been associated with so-called slow slip events in subduction zones^6^,^7^. LF events also known as long-period (i.e., dominant low frequency component in the energy spectrum) and very long-period events, are observed on all types of active volcanoes, often in swarms preceding eruption. Such LF events differ from Volcanotectonic seismicity (VT) which exhibit more typical high frequency (HF) characteristics in terms of both their characteristic frequency range and extended harmonic (coda) signature^4^,^6^,^7^ and have been postulated to be generated from fluid flow and resonance in fractures and conduits. Finally, a third type of seismicity shows features of both high frequency seismicity and also LF harmonic tremor. These so-called “Hybrid” events are characterized by a high frequency, VT-like onset and a LF-like coda, suggesting that hybrid generation is stimulated by stress regimes leading to both rock failure, and also where fluids are present in order to generate LF and tremor^4^,^5^,^6^,^7^,^8^. However, exactly how the brittle failure induces (or otherwise) secondary perturbations with LF signature has yet to be fully addressed, and a detailed analysis incorporating the coupling of these two states is missing.

Laboratory manifestations of LF, VT, and hybrid seismicity–the laboratory analogue of seismic events in Earth’s crust and a commonly-used proxy in laboratory rock physics^9^–are accessible by recording the Acoustic (phonon) Emissions (AEs). Benson *et al*.^9^,^10^ successfully recorded regular, LF, and hybrid events through state-of-the-art experiments in which acoustic excitation signals were recorded by ultrasound sensors placed on basalt rock samples during triaxial deformation experiments under dry and wet conditions. The high resolution and sensitivity of sensors used to record AEs allows for detailed study of crack tip evolution over durations of less than tens of nano-seconds at spatial scales as small as the vicinity of the crack tip. In particular, the robust nature of collective excitation modes (such as ultrasound emissions) very close to the source and prior to effects of the much faster scattering process is the major advantage in studying dynamics of the system.

In this study, by re-analyzing multi-array ultrasound excitations of hybrid events from the experiments of Benson *et al*.^9^ and comparing with their dry counterparts, we elucidate the detailed dynamics of fluid-solid micro-interaction during these experiments. We show that hybrid events are a combination of two types of cracking modes that are generated through either a “crack-like” and “pulse-like” excitations. The key difference between the two is that the pulse-like rupture is self-healing, resulting in a caterpillar or wrinkle-like rupture movement^11^,^12^,^13^,^14^,^15^. We illustrate how an intermediate phase (analogous to a “fast healing” process) in the VT section of emitted waves plays a crucial role in inducing a secondary weakening perturbation. The secondary perturbation is accompanied by a rapid decrease in pore pressure (decompression), resulting in locking of the micro-fault, analogous to mechanisms hypothesized by Lykotrafitis *et al*.^12^ and briefly by Marone & Richardson^13^. Furthermore, by mapping multi-array recorded acoustic-phonon excitations on to a fictitious spin-like system for a given cracking event, we present a counterpart of the evolution of this intricate system in the context of ordered-disordered phase transitions. Such phase transitions have been postulated in transition of crack velocities or structural phase transitions in laboratory faults^16^,^17^,^18^,^19^,^20^. Using this strategy, for the first time, we discover that an oscillatory microscopic squeezing of liquid layers imprints relatively weaker and longer disturbances of the elastic field; a key characteristic of the LF events.

## Results

**Figure 1:**
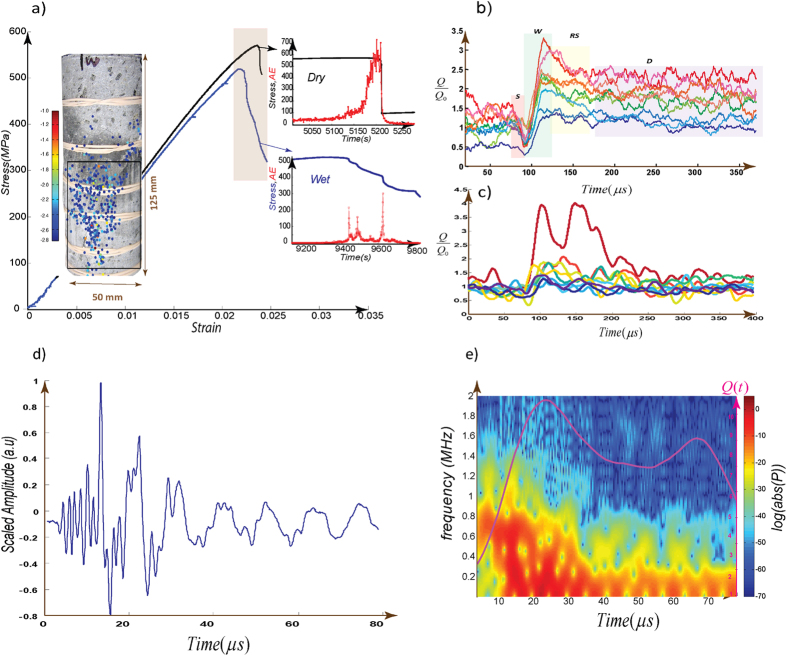
Study of Q-profiles from microscopic dry and wet events. (**a**) The strain-stress curves for both dry (black) and saturated (blue) samples. The left inset shows the water saturated sample, with AE locations with sources colored according to maximum recorded amplitude^9^. The right insets show the acoustic emission hits per seconds (red) superimposed on stress change (blue) versus time. The evaluated acoustic emissions in this study are from these intervals. (**b**) Typical acoustic emission events from dry basalt experiment. The fast weakening phase (W) leading to peak-like signal is followed by a sudden declining phase consistent with re-strengthening (RS) with a characteristic duration of ~20 ± 3 μs for typical dry events. At each excitation level and for short term evolution, *Q* asymptotically approaches a constant value (Fig. S2). For some of the events, *Q(t*) shows a slow increase during the D-phase for *t* > 180 μs. (**c**) 10 normalized Q-profiles from water-saturated basalt experiment from different stages of the experiment. (**d,e**) A hybrid event with associated spectrogram and superimposed Q(t). *P* is the Power Spectral Density (PSD) of the waveform and onset of W-phase coincides with the broadening of the power spectrum (Figs S4–S6). An overlapped (80%) 2,048-point fast Fourier transform is used to calculate the power spectral density.

We use microscopic cracking excitations recorded by multiple sensors during the rock deformation tests described extensively in ref. 9. The datasets consist of two rock deformation experiments performed on samples of Mt. Etna basalt under both water- (i.e., wet), and gas- (i.e., dry) saturated conditions (Figs 1a and S1). Both datasets were generated via conventional triaxial deformation experiments, with a suite of 12 sensors arrayed around the test samples to detect AE events. The unsaturated (dry) experiment was performed with an effective confining pressure of 40 MPa, and the saturated experiment was performed under constant pore fluid pressure (de-ionized water) of 20 MPa maintained by means of two servo-controlled pore pressure intensifiers. In both experiments a rubber jacket separates the rock sample from the confining medium (silicone oil). A confining pressure of 60 MPa was used for saturated experiments in order to yield the same effective pressure of 40 MPa for both tests. The samples in both experiments were deformed at a constant axial strain rate of 5 × 10^−6^ s^−1^, controlled via linear variable displacement transducers.

To analyze the recorded multi-array acoustic signals (Fig. 1a insets), in addition to frequency analysis, we use an innovative dynamic network-based algorithm which maps the sensors and their interactions for each recorded point on nodes and calculates the strength of similarity between nodes as “links” (Methods). We employ this class of time-series analysis together with a previously developed algorithm for multi-array records of AEs (see Methods for details of the algorithm and other techniques). The AE time series recorded at each sensor is represented as a node, and the connections between nodes (i.e., links) in each given time step are then quantified by calculating the similarity measure of the amplitudes. Then, the state of the system (source complexity, scattering with environment and instrument response) in each time-step is mapped to a network structure where the number of nodes (defining the size of the system) is held constant and the structure of the obtained network is controlled by the links. Any valid metric to evaluate an emitted phononic excitation must carry the information regarding both the source and the medium through which the phonons travel. According to network theory, for any network, a collection of metrics can be used to characterize the state of the network, and these metrics can provide information about the evolution of both the source and the medium. In this paper, we focus on two main parameters: (1), the temporal evolution of the average number of links to any one node (also referred to as the “degree” of the node: *k*_*i*_). The degree *k*_*i*_ of the *i*^th^ node at a given time represents the number of connected links to the node where “links” are established based on a similarity metric and (2), the “modularity”, an index of the community structure of the network as the “bulk” measure (see Methods). The network’s modularity characteristic is addressed as the quantity of densely connected nodes relative to a null (random) model. Generally for the studied acoustic-networks the reciprocal of the mean value of degree <*k*>, taken over all nodes, shares its main phases with the modularity, indicting the clusters of the constructed networks were modulated by the global links (Figs S2 and S3). We can understand the physical manifestation of the constructed networks in terms of *Nye-Berry’s theory* on “defects” in wavefronts^21^ which states that phase singularities at a certain point in a wavefront (i.e., the surface of constant phase) is in analogy with dislocations in crystalline materials. When mapped into a binary spin-like system, the linked nodes form a surface with the same sign as the evolution rate of each node’s degree (k). This can be interpreted as the surface of constant phase in analogy with wavefronts, and defects of this surface manifested in binary network structure as the flipping the node (spin) leading to formation of a kink (or domain wall^22^) (Fig. S14).

The first intriguing result of the analysis is that we find that ultrasound excitations possess patterns of temporal evolution of network parameters that are universal among recorded events^22^,^23^,^24^,^25^. The appearance of universal patterns in any measure of excited signals shows the robustness of the collective process in the source(s) against the much faster scattering processes. The network modularity (Q), a parameter that we describe as a “*Q-profile*” when plotted vs. time, is a measure of the temporal evolution of acoustic networks. For typical excitations, this evolution can be broken down into four main phases (Figs 1b and Fig. S2). We refer to the first short phase as the (1) S-phase: an initial strengthening phase; (2) the W-phase is a fast-slip or weakening phase; the fast weakening phase (W) leading to a *peak-like* signal is followed by (3) a sudden declining, or re-strengthening phase, (RS) with a characteristic duration of ~20 ± 3 μs for typical dry events; and finally (4) the D-phase: a slow decaying phase in long-term^22^,^23^,^24^,^25^. The origin of the RS phase has been postulated to be related to lattice cooling^23^,^26^. The long-term evolution of the D-phase is strongly coupled with scattering properties of the medium (here, the rock mass) and the magnitude of the source (which is encoded in peak of Q) but short term evolution of this phase remains another issue to be solved. At each excitation level and for short term evolution, *Q* asymptotically approaches a constant value. For some of the events, *Q* shows a slow increase in the D-phase for t > 180 μs (Fig. 1b). These main phases mirror the path of the system toward (thermal) equilibration and show how the system relaxes after a sudden perturbation. From this perspective, the system relaxes in multiple stages and time scales rather than single time scale. In general, the aforementioned phases are typical for dry events and simple friction tests conducted under dry conditions^22^,^23^,^24^,^25^,^26^ (Fig. S2).

Further analysis of acoustic networks shows an intriguing “*pairing*” mechanism, in which *kink-pair* like structures (i.e., defects in the network structure) form in the network parameters, governing the oscillations of Q(t) or other relevant parameters^23^. Furthermore, comparing the trend of Q(t) with the power spectral density (Figs 1e and S4–S6) shows that the onset of the W-phase coincides with a dramatic shift in the dominant frequency. Evaluating the path of the system in emitted signal energy versus <k(t)> space reveals a rapid change in the signal power indicating the system crossing into another energy level (Fig. S4). The results indicate that increasing <k(t)> coincides with sweeping from a lower energy level to a higher energy level. Similar multistages relaxations have been reported in recorded dynamic slip profiles and stresses measured in rock friction experiments (Fig. S2)^24^,^26^,^27^,^28^,^29^.

**Figure 2:**
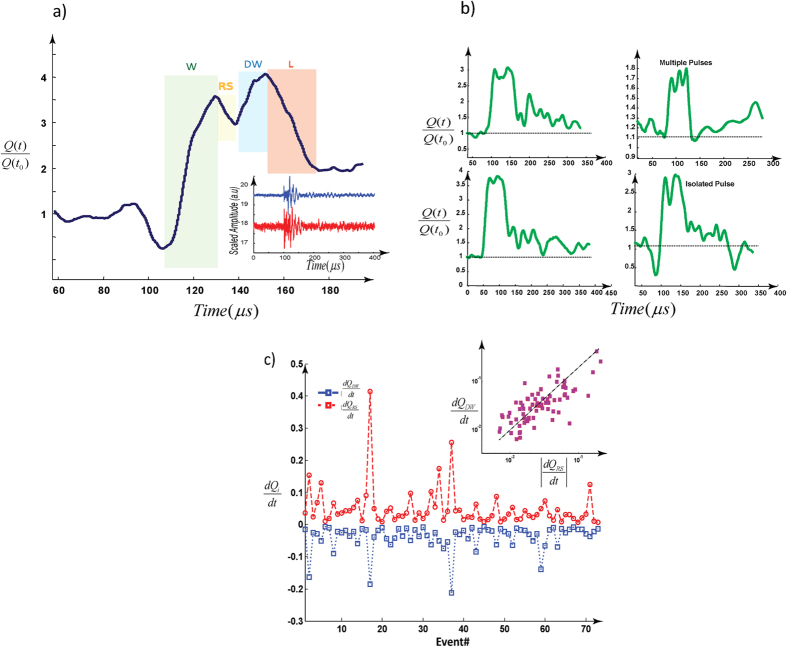
Examples of acoustic emission events with the signature of the secondary instabilities. (**a**) A typical wet-cracking noise and corresponding recorded waveforms. RS-regime is shorter than dry cracking phase and the rate of DW is significantly slower than W-regime. “L” regime is faster-than RS rate and might lead to locking the interface (see more examples in panel (b) Figs S8 and S9). (**b**) Double or multiple-weakening in wet-cracking noises. The dotted lines are the rest value (normalized) of the Q(t). (**c**) The rate of the re-strengthening regime controls the rate of secondary weakening. Events with faster RS-regime induce faster secondary weakening, well described by a power-law relation: 
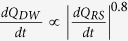
. This is based on 70 typical wet acoustic emission events (Table S1).

In contrast with typical dry cracking events in which the rate of weakening during the *W*-phase is higher than re-strengthening during the RS-phase, the recorded events under saturated conditions imprint a significantly different shape (Figs 1c and 2): For wet events the re-strengthening rate during the *RS*-phase is roughly equal to the rate of change during the W-regime, and the net-change of Q(t) approaches zero in a very short time. This is analogous to locking the crack and generating a pulse-like rupture^11^,^12^,^14^,^15^. In contrast with the dominant evolution of dry events, the RS phase is truncated by another phase, reversing the declining trend of the Q-profile which shortens the duration of the RS phase (Fig. 2a,b). This trend reversal corresponds to a secondary weakening (double-weakening, DW) phase, superimposed onto the regular “dry” profiles. The DW phase is followed by a rapid decline (L-phase) where Q(t) approximately approaches its initial value prior to the perturbation (Figs 2b and S8,S9). The rapid decline is reminiscent of pulse like cracks^12^,^13^; however, as we will see the mechanism controlling this pulse-like relaxation is unique to hybrid earthquakes.

**Figure 3:**
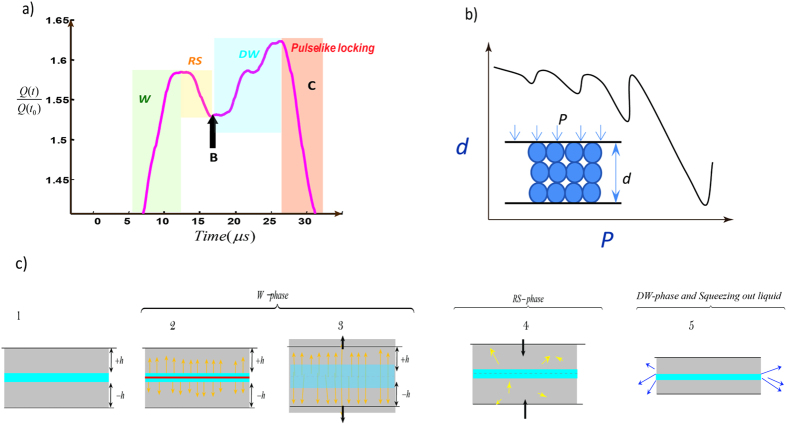
Double-Weakening Mechanism and fast-release of pore water pressure. (**a**) Fast-weakening phase is followed by a re-strengthening phase (“RS”) which leads to an increase in pore water pressure in the process zone. This results in a secondary perturbation front (“B”) with slower rate of weakening than the W-stage (DW). Then, pore pressure rapidly decreases behind the rupture tip, causing a faster re-strengthening regime and a *locking* of the micro-fault (“C”). The final shape of Q is a *double impulse* profile whereas single *peak-like* Q(t) characterizes typical dry excitations. (**b**) A schematic of liquid layers undergoing squeezing with increasing average pressure of P^30^,^31^,^32^. The variation of the pressure during squeezing as the upper block moves toward the substrate and results decreasing the number of layers. Each layer expelled results in rapid shifts in d-P space. (**c**) Schematic evolution of a portion of a “*process zone*”. The fracture energy (as the source energy) is deposited within a thin uniform layer of thickness 2 h (stage 2). Absorption and diffusion of quasi-particles in this stage results cooling-like process (stage 3). In the re-strengthening (RS) phase, contraction occurs (stage 4) which yields contraction of water layers and squeezing-out (stage 5). This leads to the DW stage and oscillatory behavior observed due to squeezing out of thin fluid layers.

We propose that the DW and L phases are related to a hydraulic perturbation and rapid release of pore fluid in the back-end of the process zone behind the propagating micro-rupture tip. The relaxation of the system due to the initial (brittle) source is modulated by the secondary hydraulic perturbation which interrupts the natural trend of the RS-phase and induces an extra weakening phase. To test this hypothesis, we examine how the onset of the DW phase becomes separated from the fast weakening (W) phase by the RS phase, and how the rate of DW is strongly correlated with the rate of RS (i.e., the faster the re-strengthening, the faster the double weakening, Fig. 2c). The observed correlation in Fig. 2c implies that the source of DW is closely coupled to details of RS evolution. Assuming that RS is the re-loading phase for the tail end of a process zone in which initial weakening took place and includes a volume of water (Fig. 3c), any change in pore-pressure in the crack is likely to be associated with the changes in RS-rate (contraction rate) and vice versa. Until point B in Fig. 3a the source behaves roughly like a dry crack, except for the relatively shorter duration of the RS phase, but at this point the fluid-solid coupling exerts a critical influence on the crack behavior. Considering that the rate of RS is ~10^1^–10^2^ s^−1^ (i.e., approaching impulsive loading) and water is nearly incompressible, the RS phase results in a rapid increase of fluid pressure in the crack and possibly localized expulsion of fluid at nano-asperities (Fig. 3c). We comment on this latter interpretation after developing the concept of K-chains below.

To explore more features of the DW mechanism, we utilize *K*-chains [22-also see section 3 of supplementary information], which help to visualize the spatiotemporal evolution of acoustic networks and shed light on the microscopic source of Q-profiles. We map the spatial evolution of the degree *k*_i_ of the *i*^th^ node (number of connected links to the node), using polar coordinates (*r*_*i*_, *θ*_*i*_)_*i*=1,..., *Nodes*_ where *r*_*i*_ = *k*_*i*_ and *θ*_*i*_ indicates the position of the node which is fixed on the outer circumference of the cylindrical sample (Figs S10–S13). To analyze the spatiotemporal evolution of *K*-chains, chains are simplified by mapping them as “*pseudo-spins*” where for each node we assign 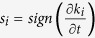 and then *s*_*i*_ = ±1. With this mapping, each node in a given time-step acquires one of the binary states (↑ or ↓). Here we use a linear *K*-chain simplification, but the true nature of K-chains are 3d where the state of each node can be represented with a 3d vector such as 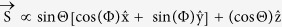 with Θ as the polar and Φ as azimuthal angles. In a linear ***x***-chain configuration, we have Θ = *π*/2, Φ = ±*π*/2 and 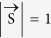. We focus on two parameters to characterize the dynamics of the chains: (1) the *“order”* parameter *m* = <*s*>; i.e., the mean of up ↑ and down ↓, where a perfectly ordered system is characterized by |m| = 1, and (2) the pair-correlation function (node-node correlation) for the system of nodes. We can fit a correlation function in the form of 
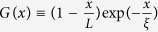
 where *L* is the total number of the nodes, *x* is distance, and *ξ* is the *correlation length*. Correlation length *ξ* is the cut-off length of the correlation function where for distances shorter than the correlation length, G(x) can be fit by a power law function. For a fully ordered state a triangular function (i.e., fully coherent system and *ξ* → ∞) is given.

**Figure 4:**
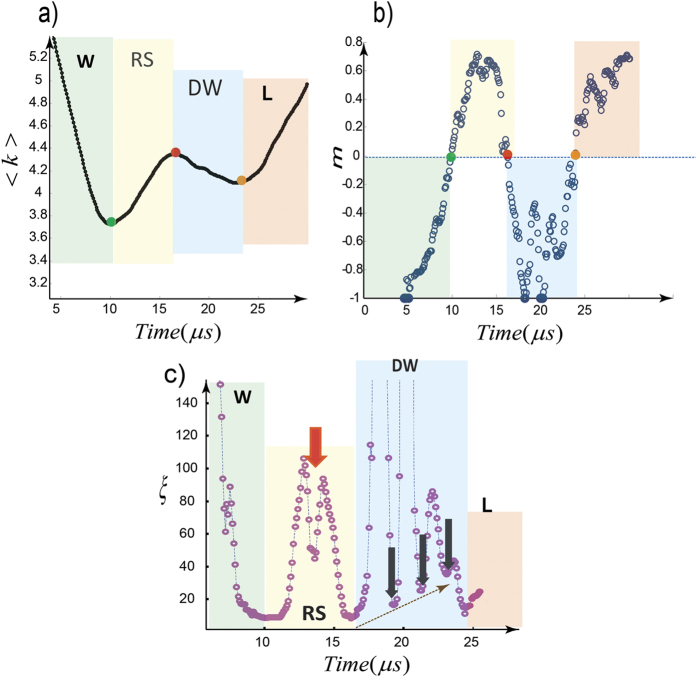
Sequences of W-RS-DW Transitions for a wet-cracking excitation with double-weakening signature. (**a,b**) The average degree of all nodes (<k>) versus time and the order parameter (*m*) with the critical transition points indicated by colored circles. (**c**) Real time node-node correlation length (*ξ*). The main transitions are indicated by *ξ(t*) minima. Highly fluctuating order parameter in DW phase drives the system in to the next degenerate state. This signature distinguishes the physics of the secondary weakening phase from the W-phase. We assign this fluctuation to discrete process of squeezing-out of the liquid (i.e., layering transition). The frequency of this almost periodic oscillation is around *200–300 kHz*. A double peak in RS phase-exhibited in *m*(t) and *ξ(t*)- induces sequence of stiffening-softening like feature with durations less than 2 μs (The system size–number of nodes–was 300 nodes–Also see Figs S15, S16 and S20).

In the transition to the DW phase, the RS-phase involves a strong spike in correlation length of the *K*-chains (Figs 4 and S15,16). A double peak in the RS phase exhibited in *m*(t) and *ξ(t*) induces a sequence of loading-unloading like oscillations, each with a duration less than *2 μs*. Another highlighted feature of hybrid events is a highly fluctuating order parameter in the DW phase which differentiates the nature of the secondary weakening phase from that of the W-phase. The secondary weakening phase is characterized by successive returns of the correlation length to local minima, suggesting rapid crossings of the critical transition points between highly metastable regimes where successive local perturbations drive the system to the next transition point. This discrete sequence increases (on average) the minimum correlation length as the system approaches the locking phase (Fig. 4). The frequency of this quasi-periodic oscillation of *ξ* in the DW regime is around *200–300 kHz* for our tests. In the context of the physical model developed in Fig. 3c, localized expulsion of fluid into the surrounding process zone of a microcrack would result in transient density fluctuation within the liquid film remaining in the crack and develop pressure differences between the “squeezed out” regions and the rest of the system. This hypothesized process is akin to the dynamics of squeezing-out layers of a liquid film under pressure^30^,^31^,^32^. The process results in decompression of water in a series of steps which is imprinted as oscillatory behavior the DW phase (Fig. 4c). Decreasing water volume and decreasing water-pressure increases the effective stress until the solid walls come into direct contact, where it is encoded as the L-phase and locking of the micro-crack^12^,^30^.

It should be noted that the vibration of the crack in the DW regime can also be modeled with Chouet’s crack-vibration formulation which was used to relate volcano-seismicity to fluid flow in fractures and conduits within a volcanic edifice^4^,^5^.

## Discussion

We quantified cracking excitations, and in the process have developed a new understanding of laboratory AEs by comparing rock deformation experiments in water saturated (wet) and dry conditions. To achieve this, we have developed the concept of *K-chains* to shed more light on micro-evolution of structures in acoustic networks. These structures reflect the processes involved in the vicinity of the propagating microcrack tips. This is crucial, as the moving crack tip generates the source of the AE energy. The introduced metrics illustrate how the system relaxes to a (thermal) equilibrium state after an excitation, and this relaxation occurs in a complex process characterized by at least three transitions (i.e., S to W, W to RS and RS to D). We demonstrated that the modulation of the relaxation path due to the secondary perturbation as a result of shortening of the RS-phase induces a secondary relaxation phase, and we ascribed this secondary relaxation state to fluid-solid interactions, in particular, the transient “squeezing-out” of thin films of water layers.

While we showed that the rapid release of pore pressure generates hybrid events in terms of mixed high and low frequencies leading to pulse-like locking of the crack, such waveforms have, in certain conditions, also been reported in dry conditions^12^,^14^. Furthermore, it has been shown that their occurrence is more probable at lower confining stresses^15^. The occurrence of self-healing pulses is typically analyzed in terms of certain conditions of state-and-rate equations in this low-stress range^15^, although reflections of waves from local heterogeneities is also thought to assist cessation of slip behind the pulse^33^. In the context of the configurations of K-chains and the observed relaxation phases, the suppression of slow relaxation phases (D-phase) will result in pulse-like excitations in which the vibrating part of the crack must be confined between the rupture front and repining (i.e., healing) point [34- see Figs S19–S20]. For a crack-like excitation the healing occurs when the system approaches thermal equilibrium^26^,^34^. We will investigate further features of such mechanisms in our future studies. Furthermore, the abstract formulation for the RS to DW transition can be extended through the addition of a poro-elastic element, and with hydrodynamic equations, to describe squeeze-out dynamics of water-layers (see Supplementary Information-Section2).

As we mentioned the coda part of the hybrid events do have the characteristics of LF spectrograms^4^,^8^. Focusing on low-frequency section of recorded waveforms (Fig. 1e), events have frequencies of ~0.5 MHz for crack length a few hundred μm. Natural volcanic low frequency earthquakes with dominant frequencies of ~0–5 Hz are associated with fracture lengths of hundreds meters^8^. Considering that dominant frequencies of earthquakes scale inversely with source dimension^10^, we have d_*l*_ × ν_*l*_ = d_n_ × ν_n_, where d_*l*_, d_n_, ν_*l*_, and ν_n_ are dimension and frequency of laboratory (*l*) and nature (*n*). Comparing our laboratory data with typical frequency (~1–2 Hz) and size (~100 m) of low frequency earthquakes, we obtain d_n_/d_*l*_ ~1 × 10^6^ and ν_*l*_/ ν_n_ ~ 0.5 × 10^6^, indicating good agreement between laboratory data and natural cases.

The role of capillary bridges, which add negative pore-pressure as the capillary bridges add an appreciable drag force^35^,^36^, has been neglected in this research. These capillary bridges act like liquid bonds, resulting in additional frictional resistance. While we considered simplified linear fictitious spin chains and analyzed corresponding order parameter oscillations, extension of these simplified K-chains to 3D K-chains will provide a unique opportunity to shed additional light on transition between phases. In particular, we hope to further elucidate the details of short term oscillation of D-phase, immediately after RS phase, which is believed to represent the slow thermalization dynamics as the unique characteristic of non-equilibrium processes^37^. Future work should also focus on the dynamics of K-chains at different ambient temperatures as large temperature gradients are a unique of volcanic seismicity in the natural setting.

## Materials and Methods

### Laboratory Procedures

The material tested is a porphyritic, alkali, lava-flow basalt from Etna volcano, Italy. The initial density of the block used in the experiments of Benson *et al*.^9^ was 2860 kg/m^3^ and the initial porosity was 3.8%. Samples of 125 mm length and 50 mm diameter were prepared using a diamond coring drill. A small conduit of 3.125 mm diameter was pre-drilled down the central axis of the sample to provide direct pore fluid access to the fault zone formed during deformation and failure of the sample^9^. This basalt was specifically chosen as previous studies have shown that it has a ubiquitous network of pre-existing microcracks. This microstructure is reflected in an anomalously low P-wave velocity of approximately 3250 m s^−1^ under ambient conditions, and a relatively high permeability (steady-state-flow with water) in the range 1 to 4 × 10^−17^ m^2^ at effective pressures from 5 to 50 MPa^38^. Cylindrical samples isolated from a confining medium (silicone oil) via an engineered rubber jacket containing inserts for mounting piezoelectric sensors in order to detect AE events-Figs S1 and S10. AE event signals (voltages) are first pre-amplified 60 dB, before being received and digitized. Standard triaxial deformation experiments were performed in which the sample is loaded at a constant strain rate until failure occurs. At this stage the fault plane, which forms typically at an angle of approximately 30°, is connected the conduit (see details in ref. 9). For water saturated experiments, an additional experimental step was carried out, in which the deviatoric stress is lowered until a hydrostat of 40 MPa effective pressure is achieved is order to ensure that no slip occurs on the fault plane.

### Networks of Acoustic Emission Waveforms

Modern complex network theory provides a powerful tool for investigating variety of complex systems^23^,^39^,^40^,^41^,^42^,^43^,^44^,^45^. Complex network theory has been employed in the analysis of time series and multivariate time series^41^,^45^, including the climate networks^42^, brain networks^43^,^44^, and multiphase flow system^40^.

To evaluate reordered multiple acoustic emission (multiple time series for an occurred event), we use a previously algorithm on waveforms from our reordered acoustic emissions^22^,^25^ which is the class of functional connectivity networks from multivariate time series data. The main steps of the algorithm are as follows:

1. The waveforms recorded at each acoustic sensor are normalized to the maximum value of the amplitude in that station.
2. Each time series is divided according to maximum segmentation, in a way that each segment includes only one data point. The amplitude of the *j*th segment from *i*th time series (1 ≤ *i *≤ *N*) is denoted by *u*^*i,j*^(*t*) (This is in the unit of m-Volt). *N* is the number of nodes or acoustic sensors. We put the length of each segment as a unit. This considers the high temporal resolution of the system’s evolution, smoothing the raw signals with 20–40 time windows (~400–800 ns).
3. *u*^*i,j*^(*t*) is compared with *u*^*k,j*^(*t*)to create an edge among the nodes. If *d(u*^*i,j*^(*t*), *u*^*k,j*^(*t*)) ≤ *ζ* (where *ζ* is the threshold level discussed in the following point) we set *a*_*ik*_(*j*) = 1 otherwise *a*_*ik*_(*j*) = 0 where *a*_*ik*_(*j*) is the component of the connectivity matrix and 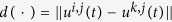 is the employed *similarity metric*. With this metric, we simply compare the amplitude of sensors in the given time-step. The employed norm in our algorithm is absolute-norm.
4. Threshold level (*ζ*): To select a threshold level, we use an introduced method in refs 23,25 and references therein] where uses an adaptive threshold criterion and is stable for an range of deviations from *ζ*. The result of this algorithm is an adjacency matrix with components given by *a(x*_*i*_(*t*), *x*_*k*_(*t*)) = Θ(*ζ*−|*u*^*i,j*^(*t*)−*u*^*k,j*^(*t*)|) where Θ(...) is the Heaviside function.

In general, the modularity of a network measures the degree of division of that network into modules: if a network has high modularity, the strength of connections in individual modules is strong, whereas the strength of connections between modules is not. The network’s modularity characteristic is addressed as the quantity of densely connected nodes relative to a null model (random model). The main diagnostic in this work is the *Q-profile*. The modularity is the result of some optimization of the cluster structure of a given network. The modularity *Q* (i.e., objective function) is defined as^39^:


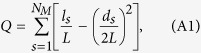


in which *N*_*M*_ is the number of modules (clusters), 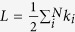, *l*_*s*_ is the number of links in module *s* and 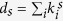 (the sum of node degrees in module *s*).

We use the Louvian algorithm to optimize Eq. A1^39^, which has been used widely to detect communities in different complex networks. Then, in each time step during the evolution of waveforms (here over observation windows of ~400 μs), we obtain a Q value. The temporal evolution of Q values in the monitored time interval forms the Q-profile.

The introduced network algorithm is in close relation with *space-correlation methods* as well as temporal frequency analysis (Figs S4–S6). We can define a similar measure to *time-widowed correlation method*^29^,^30^ where the inner product is replaced with Heaviside function. Let us consider a sequence of nodes in a certain time step and the length of this sequence is L = 2 *l* and the amplitude of each node is *u(x*). The space windowed correlation is given by^46^,^47^:


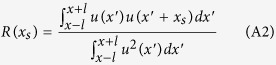


where the space-window is centered at length *l* with duration 2 *l* and *x*_*s*_ is the space shift used in the cross correlation. Here the employed norm is inner-product and can be replaced with another norm:


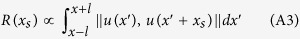


Summing over all space-shifts and replacing the norm with a similarity metric we get:


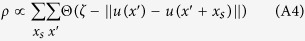


Then, *ρ* is proportional to the density of links owning to the employed metric where we use to construct the links between nodes represented by pairs of *u(x*′), *u(x*′ + *x*_*s*_).

Furthermore, we understand the physical manifestation of the constructed networks in terms of *Nye-Berry’s theory* on “defects” in wavefronts^21^. The theory states that phase singularity or nodal line at a certain point in a wavefront (i.e., the surface of constant phase) is in analogy with dislocations in crystalline materials. On these defects, the wave amplitude is zero. When the introduced acoustic-networks mapped into a binary spin-like system 
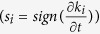
, the linked nodes form a surface with the same sign as the evolution rate of each node’s degree. This can be interpreted as the surface of constant phase (or same family of rays) in analogy with wavefronts, and defects of this surface manifested in binary network structure as the flipping the node (spin) leading to formation of a kink (or domain wall^22^) in K-chain (see Fig. S14). From this perspective, evolution of different relaxation stages after splitting a matter-interpreted with Q(t)- is understood as the creation and annihilation of phase singularities of wavefronts.

## Additional Information

**How to cite this article:** Ghaffari, H. O. *et al*. Microscopic Evolution of Laboratory Volcanic Hybrid Earthquakes. *Sci. Rep.* **7**, 40560; doi: 10.1038/srep40560 (2017).

**Publisher's note:** Springer Nature remains neutral with regard to jurisdictional claims in published maps and institutional affiliations.

## Supplementary Material

Supplementary Information
