# Supplementary material for: Microscopic Evolution of Laboratory Volcanic Hybrid Earthquakes
Source: Sci Rep. 2017 Jan 11;7:40560. doi: 10.1038/srep40560 (PMC5225436; doi:10.1038/srep40560)
Supplement: Supplementary Information [file srep40560-s1.pdf]

Supporting Information for

Microscopic Evolution of Laboratory Volcanic Hybrid Earthquakes

H.O. GHAFFARI [1(A)], W.A. GRIFFITH[1] , P.M. BENSON [2]

1 Department of Earth and Environmental Sciences, University of Texas at Arlington, Arlington, TX, 76019, USA
2 Rock Mechanics Laboratory, University of Portsmouth, Portsmouth , UK

General Points of the Supplementary Information

In this Supplementary Information, we provide further results and discussions on the proposed ideas in the main text.

- The datasets were recorded at different loading stages of our tests.
- The details of the acquisition system (ASC system) and the method employed to analyse acoustic excitations and were reported in earlier publications (Benson et al 2010; Benson et al 2008). The schematic picture of the experimental set-up is shown in Fig.S.1.
- The duration of the RS-phase and hydro-mechanical formulation of RS-DW phases
- K-chains: further examples and mapping near-field acoustic excitations to spin systems

1. Some general points on experiments

1-1-

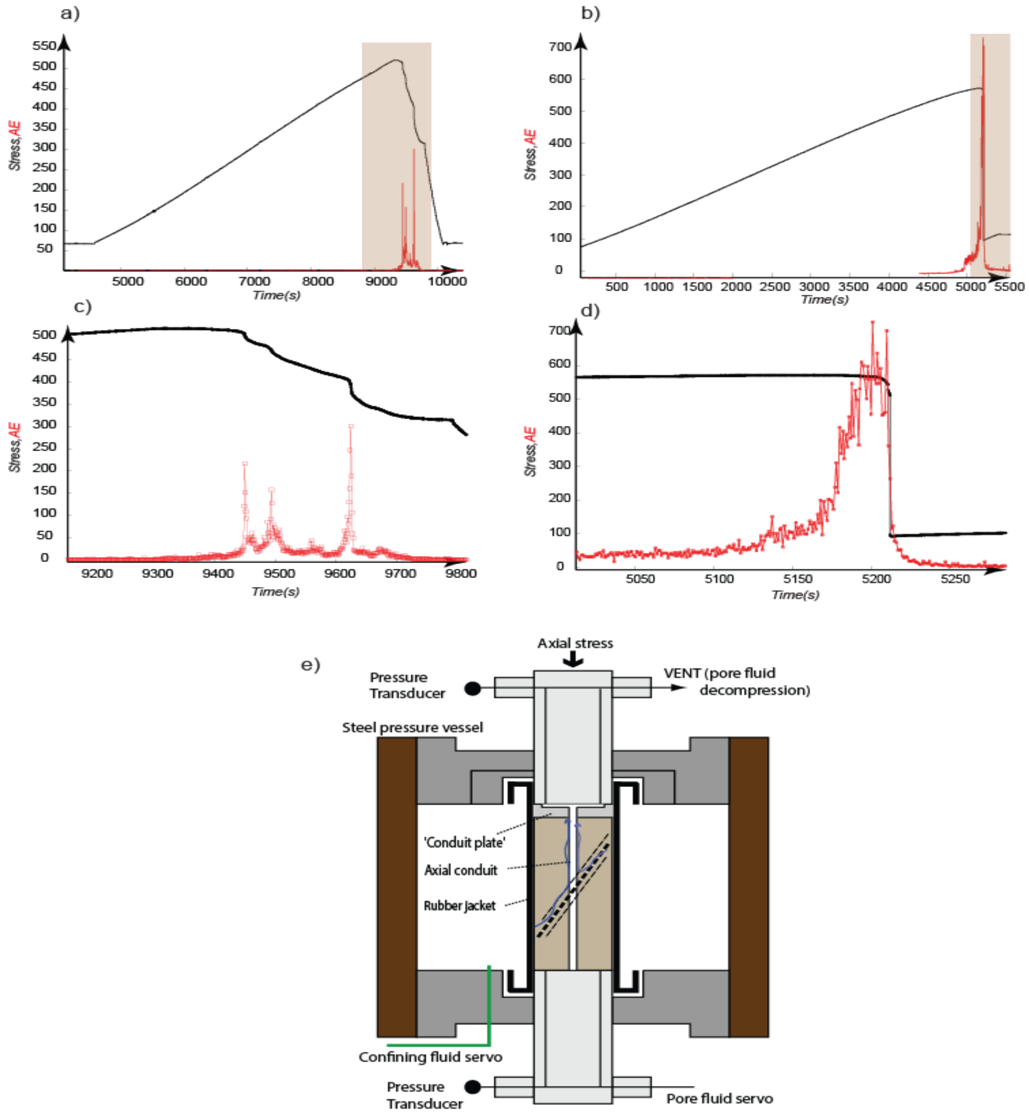

Figure S.1 (a-d) acoustic emission as the number of hits per second is superimposed on the stress-time curves. Time is the global time of the tests (loading instrument time) in seconds. The evaluated events for both and wet are from the highlighted interval. The rate of microcracking during loading is indicated by the AE hit rate, defined as bulk AE "hits" averaged over the 16 AE sensors per second, with a "hit" defined as every instance that a pre-set number of sensors (6) register voltage above a set threshold (60 mV). (e) A schematic picture of Experimental setup [modified from (2)]. Standard triaxial deformation experiments were performed in which the sample is loaded at a constant strain rate until failure occurs. At this stage the fault plane, which forms typically at an inclination angle of approximately 30°, is connected to the conduit (see details in Benson et al. 2010). For watersaturated experiments, an additional experimental step was carried out, in which the deviatoric stress is lowered until a hydrostat of 40 MPa effective pressure is achieved is order to ensure that no slip occurs on the fault plane.

1-2-On dry micro-acoustic ultrasound events- To analyze the recorded micro-excitations, we use the relations between different phases as defined in terms of network parameters (Fig. S2a,b). The dry events are mapped to $\frac{dQ_w}{dt} - R_D$ space, where $R_D \equiv Q_D^{-1}$, which displays a constant exponent in the power-law fitted function : $R_D \propto (\frac{dQ_w}{dt})^{-\gamma}, \gamma \approx 0.7$ (Fig.S2c). Assuming that the *D*-phase represents the nearly steady-state regime in comparison with the rate of the preceding phases, we interpret this parameter space as the bridge between the fast weakening rate (i.e., fast relaxation state) and steady-state stress: faster weakening rate yields smaller R_D (Fig. S2c). Therefore, we infer a microscopic weakening law where steady state resistance (i.e., *R*) is scaled inversely with weakening velocity.

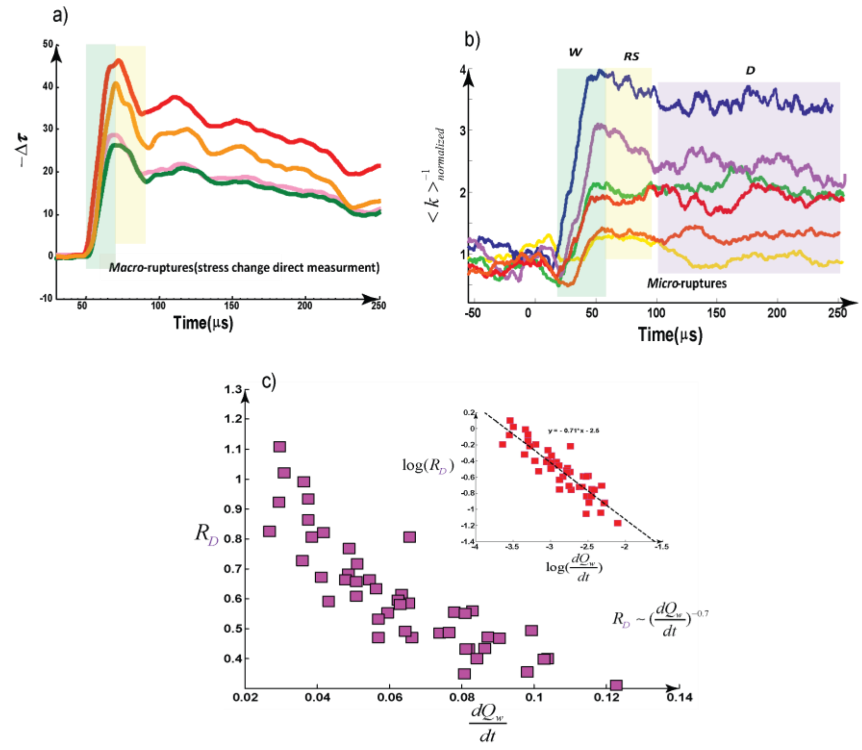

Figure S.2 **<u>Dry events:</u>** (a) the measured dynamic shear stress change using (dynamic) strain gauge while two halves of the saw-cut Westerly granite slide on each other [5-6 ;supplementary Information of Ref.6] leading to macro-slips. We show four stick-slip events. The fast weakening phase is shown with the green rectangle. Wheatstone bridge strain gages are employed to record the dynamic (shear) stress change at 10MHz which is mounted at 7 mm from the saw-cut fault (the slope of the fault was 60' with horizon –Also see Thompson et al. experiments [Thompson, B. D., R. P. Young, and D. A. Lockner (2005)-Ref.9] (b) Inverse of the mean degree (<k(t)>) profiles from <u>micro-cracking</u> excitations (c) Rate of W-regime approximately scales inversely with the steady-state value of D-phase in terms of Q^{-1} (i.e., $R_D = Q_D^{-1}$) as it occurs approximately in D-phase: $R_D \propto (\frac{dQ_w}{dt})^{-\gamma}, \gamma \approx 0.7$ The events are from the dry experiment. This observation results in a microscopic weakening law where steady state resistance scales inversely with weakening rate.

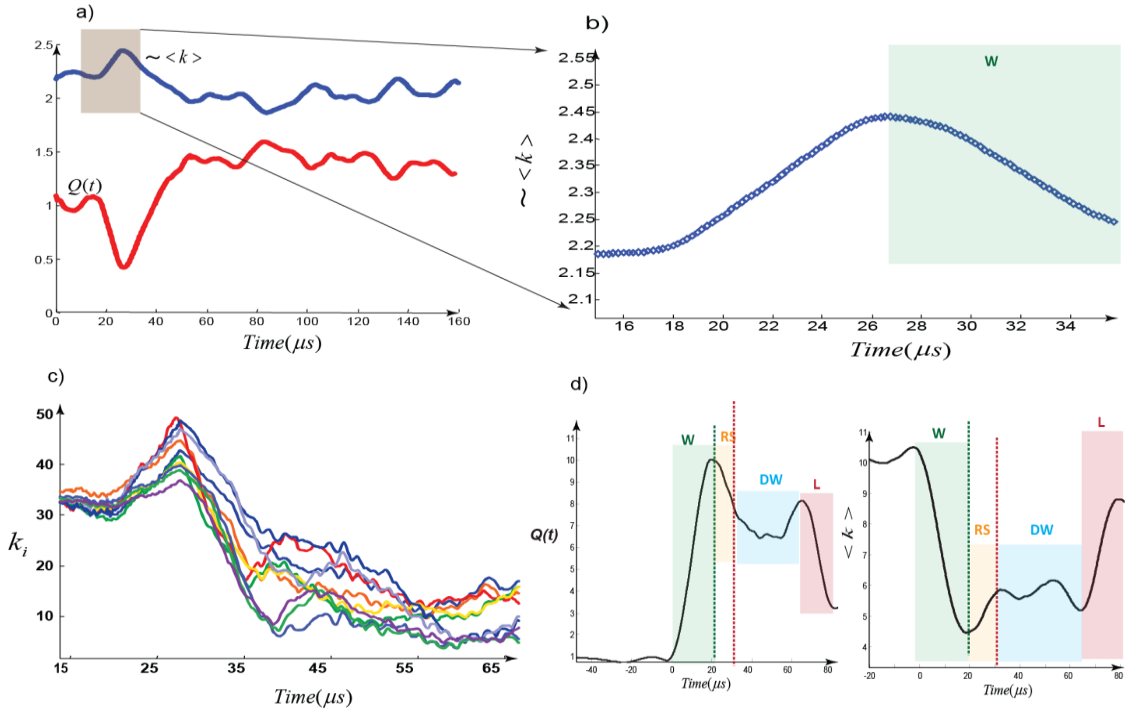

Figure S.3| a) Evolution of mean degree of the constructed network and the modularity index (Q(t)); b) evolution of scaled <k> . (c) We show the evolution of <k> for a dry event. (d) Wet-event: normalized Q(t) and <k(t)> with W-RS-DW-L phases. We conclude that for our network: $Q(t) \cong < k >^{-1}$ and therefore both can be used as proxies for strength evolution as per [5,6].

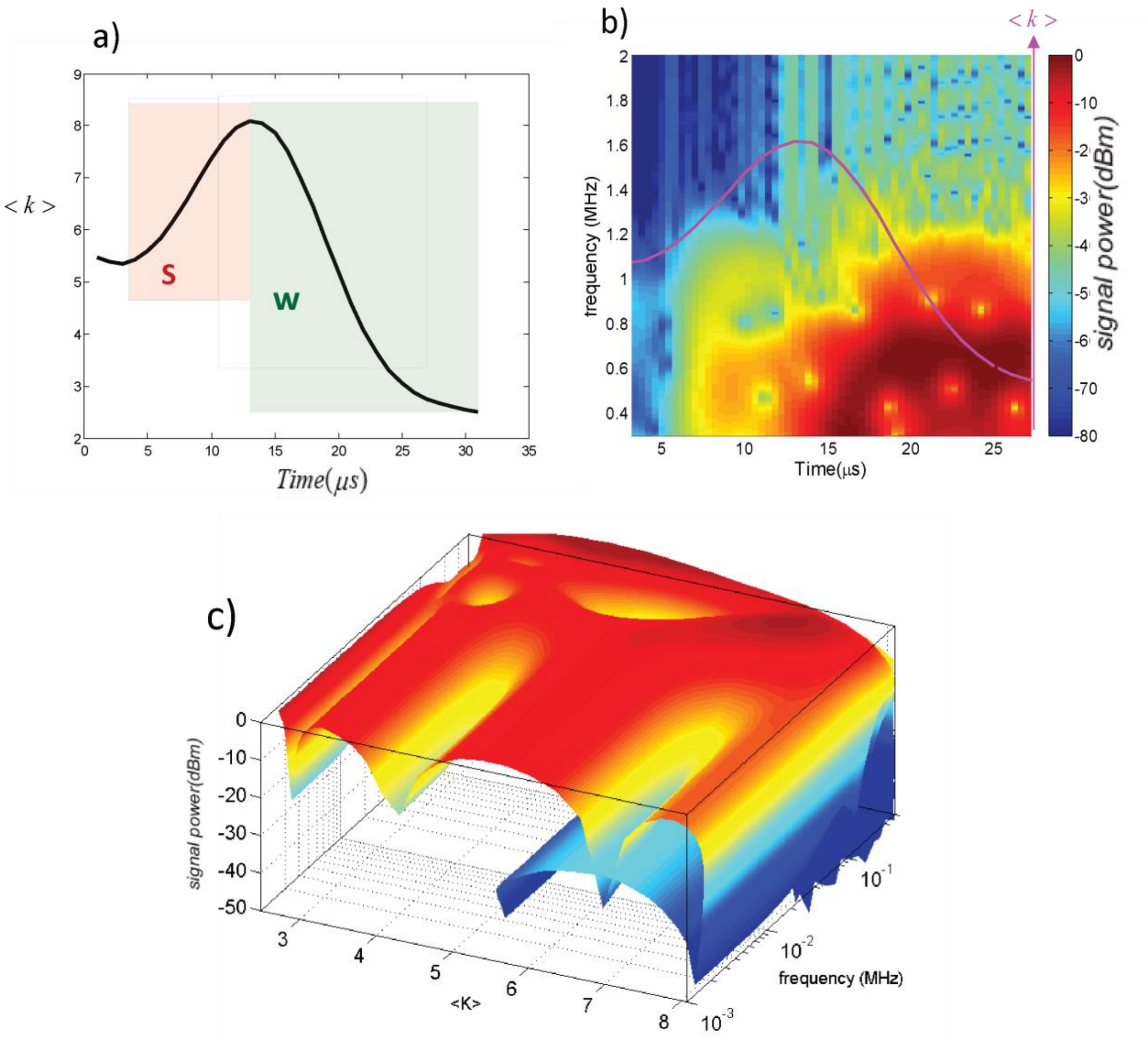

Figure S.4 **<u>Dry event</u>**:(a) Fast crossing from the S to W phase in <k>-time space .(b,c) The transition from S to W is reflected in <k(t)> which is clearly related to a transition to another energy level. In (c), we show the 3D representation of *signal power-frequency-<k(t)>*. The S-phase coincides with the lower signal power and the transition to W-phase occurs rapidly.

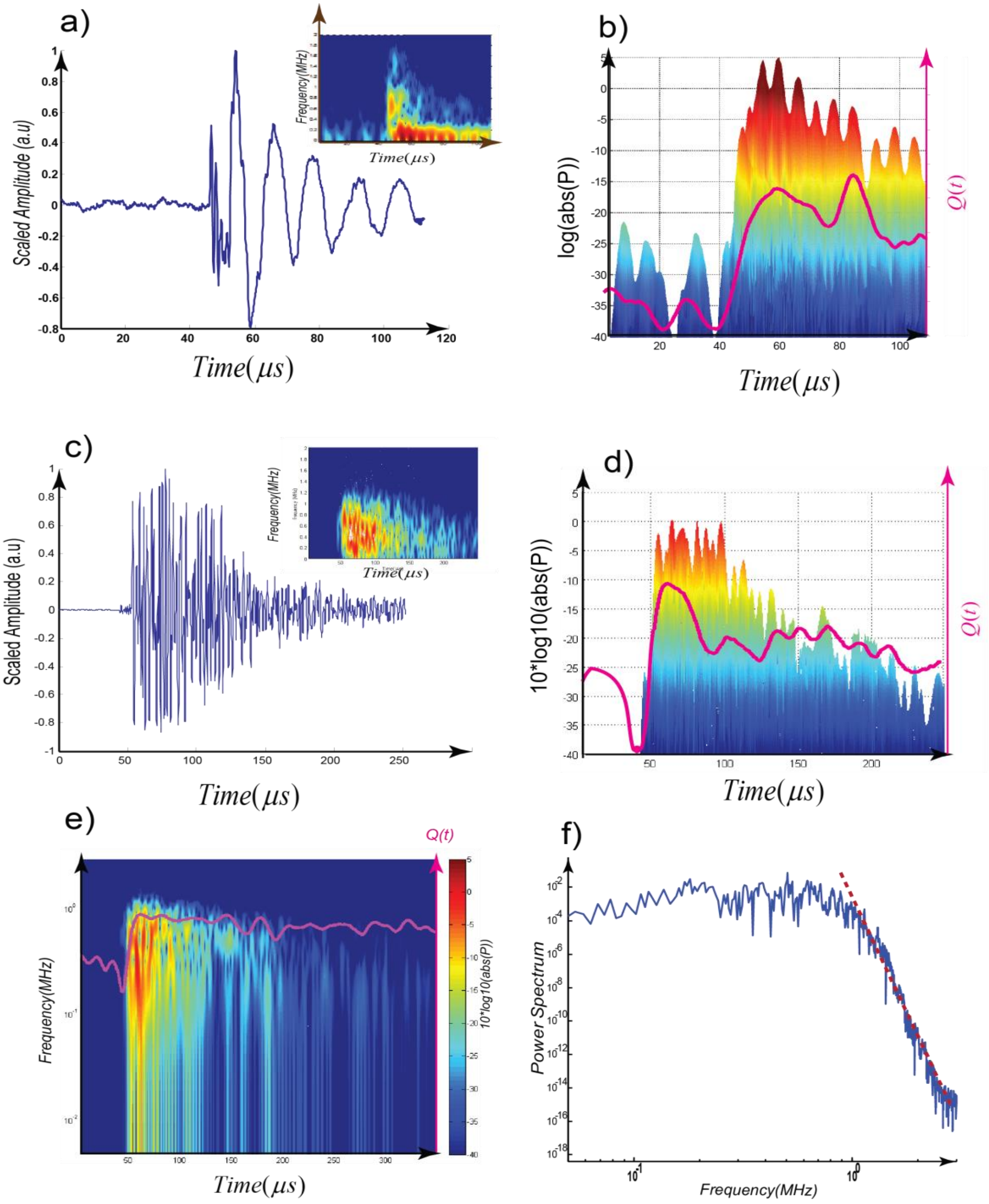

Figure S.5 Selected **Hybrid (a,b) and Dry (c,d)** events with associated spectrograms and Q(t). The insets show the spectral amplitudes versus frequency and time. With this comparison, we find that the onset of the high-frequency regime corresponds to with the W-phase in Q(t). P is the power spectral density. The onset of the W-phase is also roughly coincident with the broadening of the power spectrum. An overlapped (80%) 2,048-point fast Fourier transform is used to calculate the power spectral density. (e,f) another example from a **dry cracking** event with the corresponding spectrum.

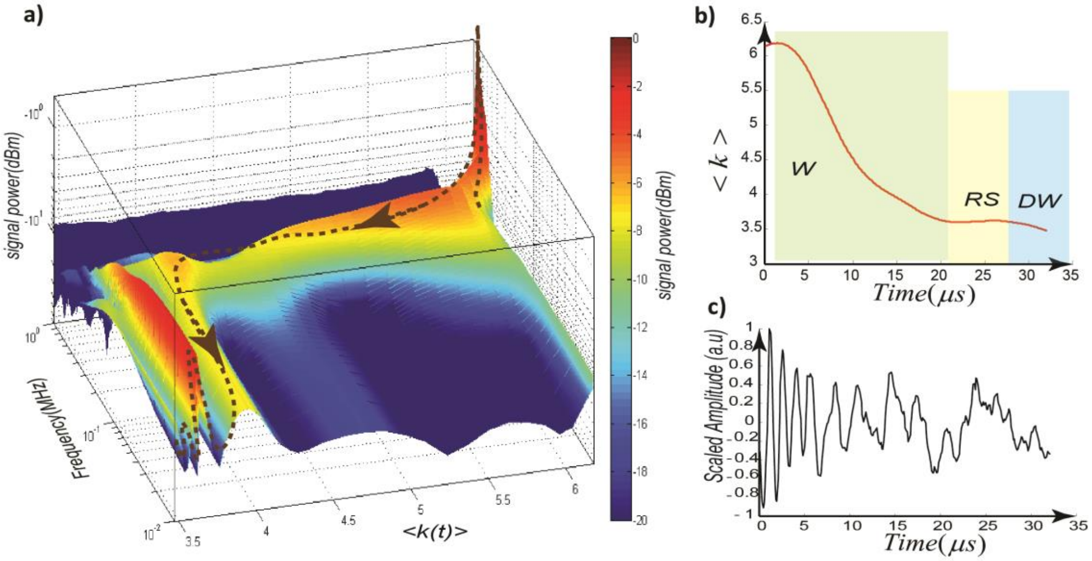

Figure S.6 **<u>Hybrid event</u>** **:** a) A 3D representation of *signal power-frequency-<k(t)>*. b-c) We show the W-RS phases and one of the waveforms (from 12 recorded signals). The result indicates higher-frequency component of waves dominates the power of the signal in W-phase, and approaching the RS phase, the lower frequency components control the path of the system.

2. Characterization of wet-cracking excitations

2-1-

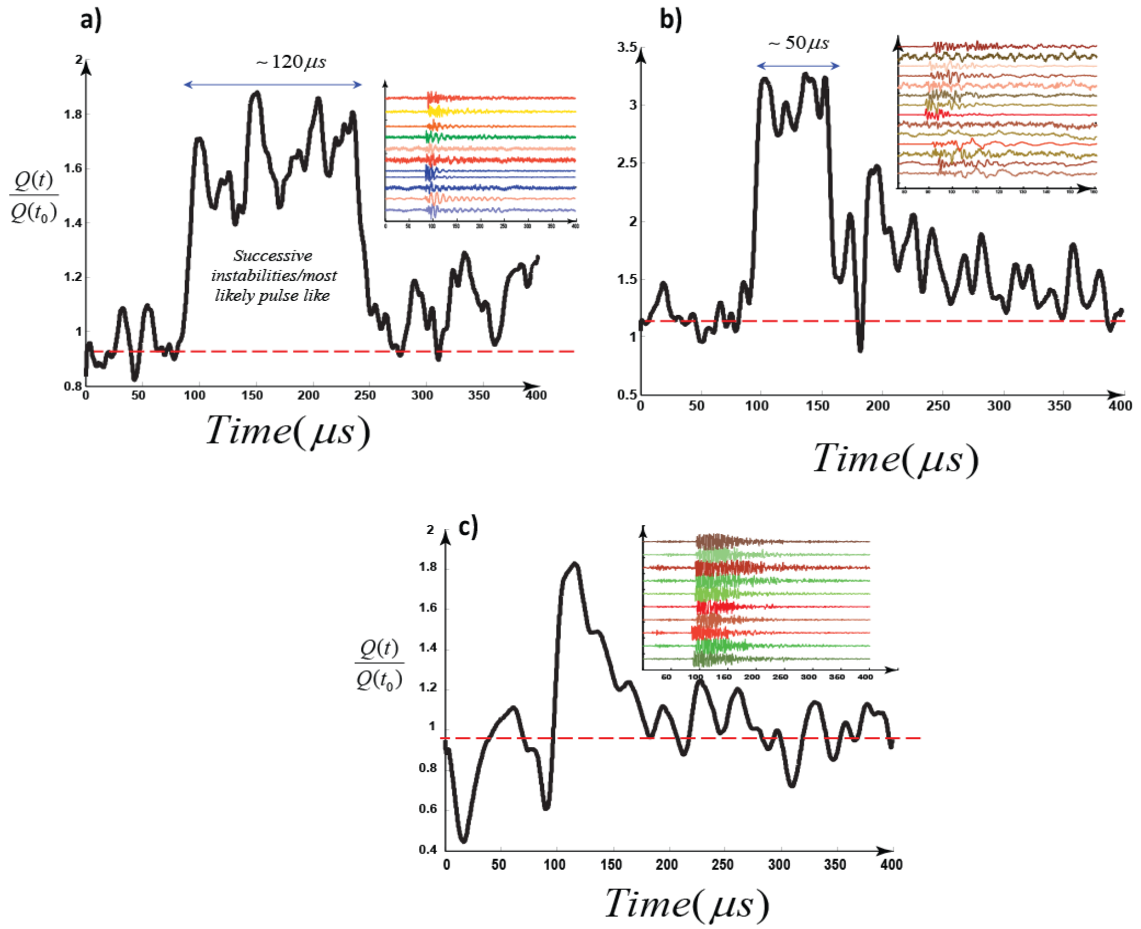

Figure S.7.*Pulse-like* ruptures (slip pulses) are frequent in "wet events". (a,b) Rapid-drop of Q(t) after the peak point indicates fast re-strengthening (locking) phase, dissimilar to typical crack-like ruptures (c). In panel (a), we show a hybrid event with a very long duration secondary instability with successive pulse-like instabilities (multiple peak-like excitations).

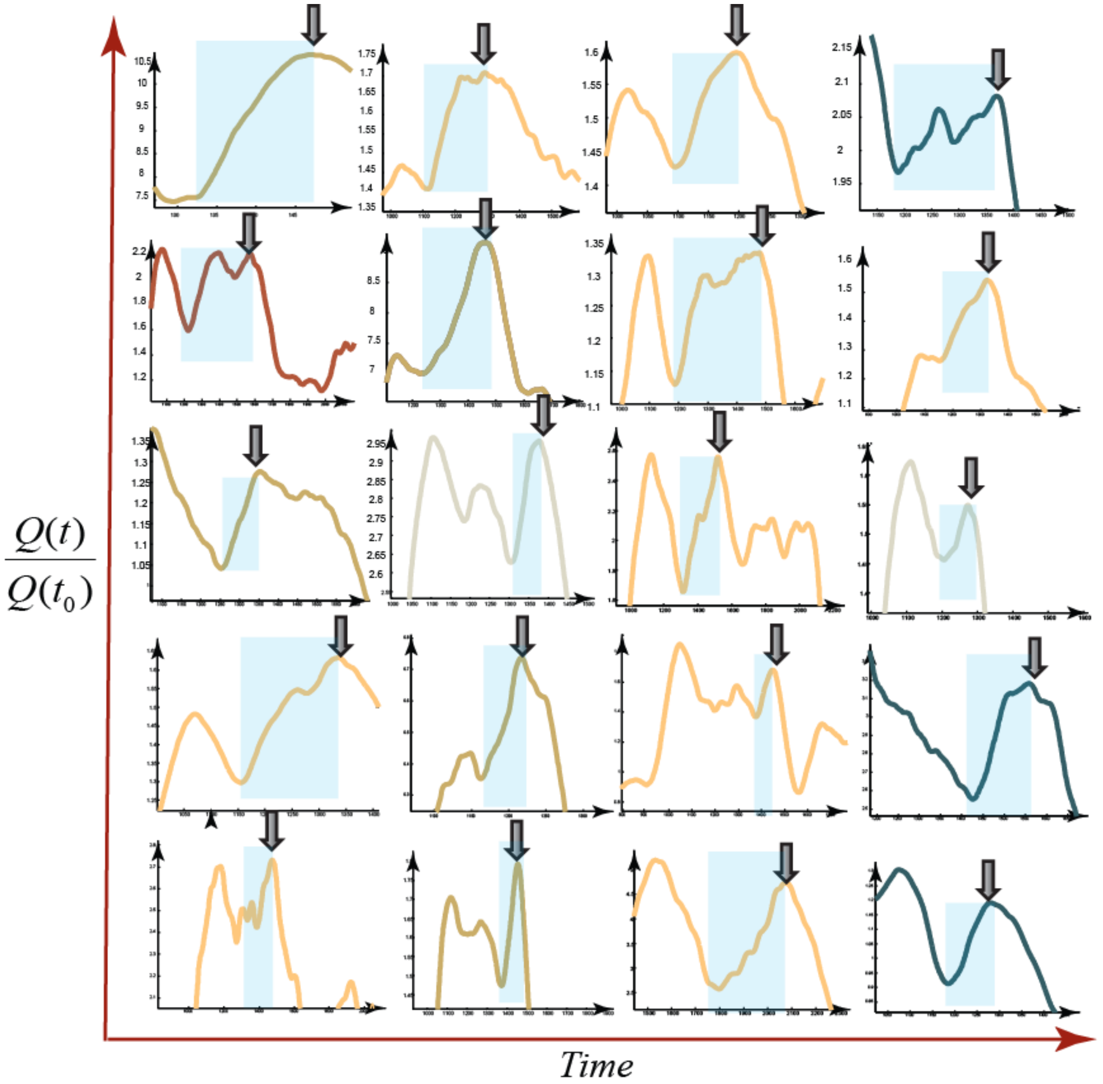

Figure S.8 Examples of secondary instability in wet events .The arrow indicates the peak of the secondary instability.

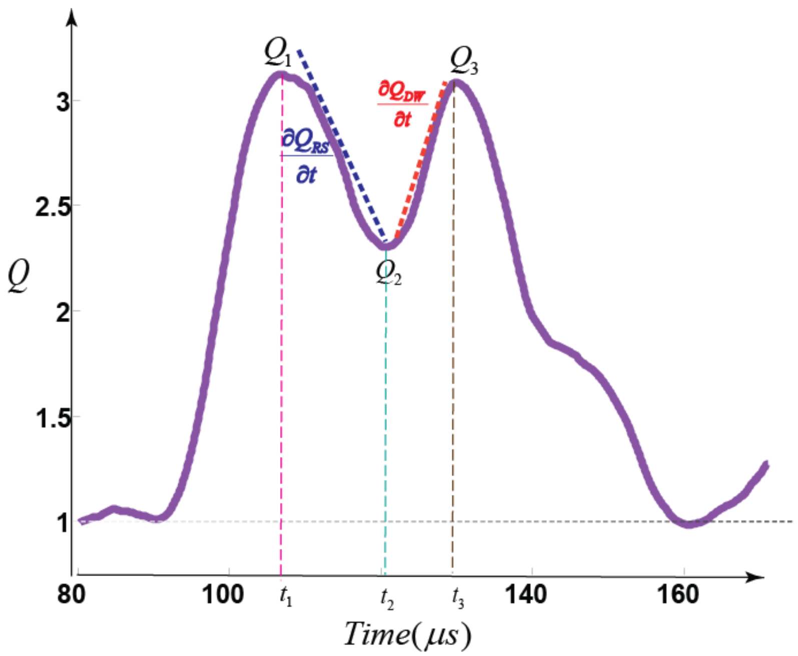

Figure S.9 A typical Q-profile with double-weakening signature as the result of hybrid events

Event#	t_1(*10)μs	Q_1	t_2(*10)μs	Q_2	t_3 (*10)μs	Q_3
1	1156	1.878	1265	1.721	1372	2.106
2	1118	10.55	1383	6.214	1576	9.171
9	1164	2.143	1266	1.895	1448	2.344
12	1139	2.157	1322	1.639	1485	2.755
13	1042	3.5331	1073	3.376	1125	4.054
15	1049	1.266	1063	1.256	1115	1.3115
17	859	1.111	922	1.051	1040	1.271
19	1085	3.004	1189	2.072	1330	3.024
26	917	2.106	1027	1.873	1150	2.277
28	920	2.109	1029	1.837	1153	2.275
38	1137	1.937	1210	1.806	1368	2.495
43	1124	2.54	1213	2.228	1279	2.509
61	1086	2.388	1145	2.264	1250	2.819
62	1055	2.917	1191	2.105	1293	2.878
68	1226	1.718	1325	1.582	1384	1.653
78	878	1.135	948	1.02	1030	1.77
81	1129	18.73	1447	12.82	1572	18
97	1129	3.146	1202	2.867	1341	3.552
104	1058	2.318	1118	2.205	1175	2.305
122	996	1.419	1102	1.309	1294	1.473
125	1045	2.808	1138	2.394	1239	2.805
126	1037	2.464	1150	1.758	1458	3.346
138	1016	2.238	1200	1.472	1478	1.917
139	1077	3.064	1103	3.028	1158	3.176
147	971	2.105	1059	1.788	1231	2.249
151	1041	1.9	1124	1.799	1272	2.339
163	1111	7.322	1191	6.935	1432	9.2999
187	1100	1.61	1196	1.454	1265	1.55
193	1056	2.024	1228	1.391	1336	1.78
195	1139	1.297	1229	1.227	1292	1.343
197	1099	2.976	1174	2.727	1216	2.879
213	1159	6.505	1184	6.347	1243	6.953

219	1084	2.934	1170	2.712	1216	2.965
224	1134	2.113	1229	1.639	1290	2.702
235	1082	3.165	1224	2.11	1662	2.702
246	1041	3.73	1071	3.569	1109	3.729
261	1069	6.734	1113	5.8	1162	7.051
264	908	1.773	976	1.613	1037	1.875
276	1137	2.294	1162	2.219	1223	2.487
311	1059	1.48	1115	1.38	1190	1.494
325	1073	3.822	1132	3.568	1182	3.687
330	990	1.123	1057	1.057	1197	1.371
335	1065	5.299	1151	4.576	1325	5.673
338	1108	1.534	1247	1.272	1339	1.426
356	989	1.496	1076	1.435	1099	1.456
357	1002	1.321	1096	1.158	1309	1.509
362	997	2.492	1058	2.372	1133	2.577
365	1053	2.456	1216	1.442	1295	2.136
374	1300	1.421	1357	1.341	1404	1.383
377	1044	1.429	1136	1.226	1193	1.326
378	1068	1.601	1218	1.167	1318	1.493
382	1047	3.564	1118	3.11	1183	3.459
387	1061	1.041	1131	0.9551	1205	1.076
413	1109	1.179	1211	1.015	1274	1.124
421	1047	2.137	1081	2.075	1148	2.363
425	1042	1.735	1109	1.536	1165	1.691
429	1129	3.309	1260	2.635	1326	2.786
431	1212	1.279	1393	0.776	1491	1.081
445	1123	5.261	1306	2.707	1566	4.021
478	1129	4.18	1224	3.561	1321	4.269
479	1047	1.432	1164	1.247	1225	1.413
482	1116	1.246	1222	1.137	1260	1.19
497	1183	6.683	1280	6.014	1326	6.224
504	1062	1.576	1133	1.478	1264	1.591
509	1052	1.519	1127	1.334	1235	1.672
510	1132	1.504	1298	1.251	1398	1.57
515	897	1.454	1004	1.372	1124	1.611
528	1107	1.361	1168	1.275	1247	1.329
541	1046	2.782	1145	2.627	1213	2.77
544	1023	1.721	1174	1.296	1256	1.567
546	1111	4.126	1151	3.976	1234	5.014
552	1073	2.689	1109	2.608	1169	2.674
558	1064	1.371	1137	1.28	1221	1.334

Table S1. List of the used Typical hybrid events plotted inFig.2(Inset). See the definition of the parameters in Fig.S9.

2-2-To proceed, we estimate the duration of the RS phase during a single hybrid event. The rate of increasing fluid pressure ($P_w(t)$) in the RS stage is proportional to the rate of re-strengthening:

$$\left|\frac{\partial Q_{RS}}{\partial t}\right| \cong k_{RS} \frac{\partial P_w(t)}{\partial t} . \qquad \text{(S.1)}$$

This statement also is true for the DW regime where at the onset of this phase the pore-pressure is in its maximum value, inducing secondary weakening, and at the end of this phase the pore-pressure approaches zero:

$$\frac{\partial Q_{DW}}{\partial t} \cong -k_{DW} \frac{\partial P_w(t)}{\partial t} . \qquad \text{(S.2)}$$

The coefficients k_{RS} and k_{DW} indicate the interaction of water and solid body in loading (RS) and unloading regimes (DW), respectively. Furthermore, we estimate empirically (Fig.2C-inset/main text):

$$\frac{dQ_{DW}}{dt} \propto \left|\frac{dQ_{RS}}{dt}\right|^{\beta}, \qquad \text{(S.3)}$$

where $\beta \approx 0.8$ (Fig.2c-main text). We can assume a linear growth of pore-fluid pressure in the RS regime:

$$\frac{\partial P_w(t)_{RS}}{\partial t} \propto t , \qquad \text{(S.4)}$$

Plugging (4) in (2) and (3) and using (4), we obtain:

$$P_w(t)_{t\in RS} \propto (\frac{k_{RS}}{k_{DW}}) \frac{t^{\beta+1}}{\beta+1} \qquad \text{(S.5)}$$

and then,

$$P_{\max}(w) \propto (\frac{k_{RS}}{k_{DW}}) \frac{t_{RS}^{\beta+1}}{\beta+1} \qquad \text{(S.6)}$$

To trigger the secondary perturbation, we should satisfy $P_{\max} \geq \sigma_{th}$ where σ_{th} is a stress-threshold quantity for the hydraulic perturbation and (6) is simplified to yield:

$$t_{RS} \geq \Theta \sigma_{th}^{\frac{1}{1+\beta}} , \qquad \text{(S.7)}$$

with $\Theta = \left(k_{DW} \frac{\beta+1}{k_{RS}} \right)^{\frac{1}{1+\beta}}$. From Fig.2C-inset (main text), $\beta \approx 0.8$ allowing a lower bound for the duration of the RS phase to be approximated as: $t_{RS} \geq \Theta\sqrt{\sigma_{th}}$. This indicates that increasing σ_{th} will stretch the minimum duration of the RS regime. In contrast, increasing β shrinks the duration of t_{RS} , leading to earlier onset of the DW phase.

3. Further characterization of excitations with K-chains

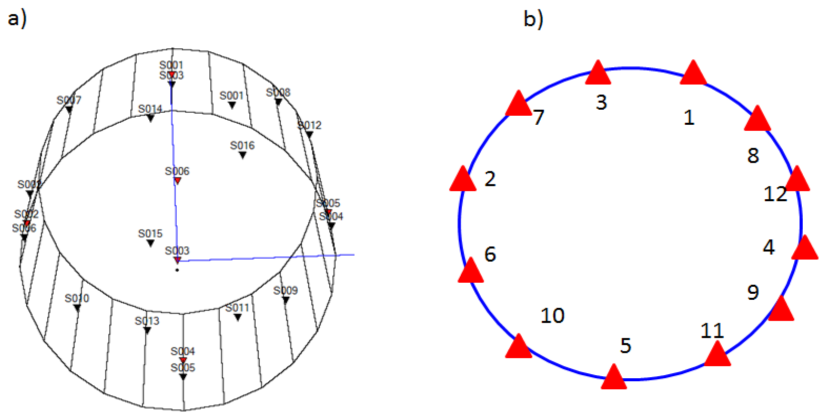

Figure S.10| Set-up of the sensors (nodes) in our tests in 3D and 2D representations.

3-1-Acoustic K-chains To shed more light on the universal network patterns of events, we visualize the spatiotemporal evolution of networks in terms of the patterns of links between nodes, where links are defined based on a similarity measure of amplitudes recorded at each sensor at the same time. This is very helpful in understanding the evolutionary phases of Q(t). In addition the trend of $R \equiv Q^{-1}$ is comparable to <*k*(t)>, the average degree of all nodes in the system at a given time. Here *k(t)* is the degree of each node, defined as the number of links connected to that node at as a function of time and <…> represents the average of all nodes. We visualize the spatial evolution of the degree k_i of the ith node (number of connected links to the node), using polar coordinates $(r_i, \theta_i)_{i=1,...,Nodes}$ where $r_i = k_i$ and θ_i indicates the position of the node which is fixed on the outer circumference of the cylindrical sample. We refer to these configurations as "*K-chains*", and the normal vector of the *K*-chains at each node indicates the local direction of increasing/decreasing k_i with time. We evaluate the variation of $r_i = k_i$ at each spatial position (i.e., each node) while we consider the temporal evolution of each single event (Fig.S10). To analyze the spatiotemporal evolution of *K*-chains, chains are simplified by mapping them on the "*spin chain*" where for each node we assign $s_i = sign(\frac{\partial k_i}{\partial t})$ and then $s_i = \pm 1$ (Fig.S10-S13). With this mapping, each node in a given time-step acquires one of the states ($\uparrow$ or $\downarrow$).

In a fully "saturated" state (all arrows direction are up or all are down; all nodes are of identical sign) and the *flipping* of nodes are represented with negative s_i indicated by

inward-pointing normal vectors (Fig. S13- Ghaffari et al., 2016 [5]). This damaged node acts similar to a kink, separating zones with up and down nodes. In Fig.S13, we have shown that creation and annihilation of kinks-antikinks (i.e., pairing mechanics) govern the transitions between main phases of Q(t) (or <K(t)>).

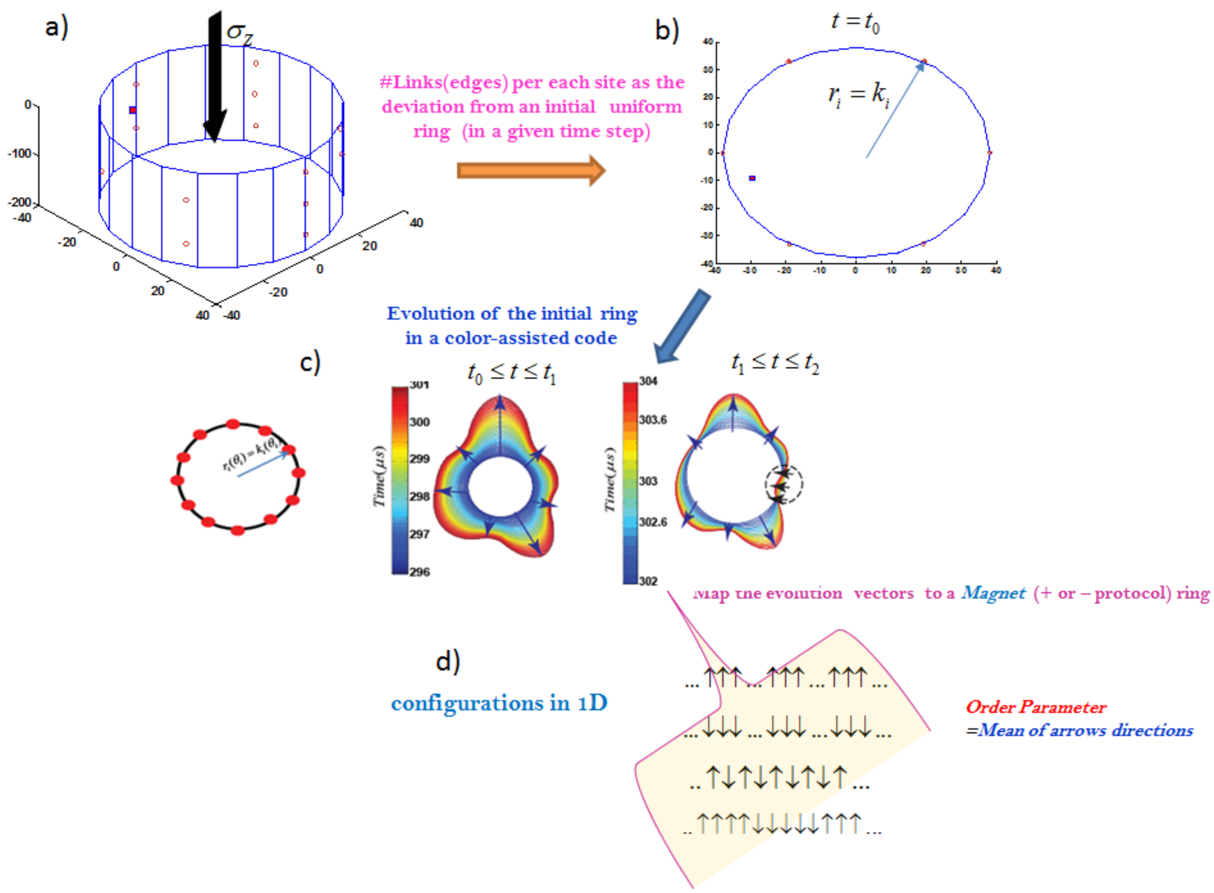

Figure S.11| (a-d) Steps to map the K-chains to fictitious spins.

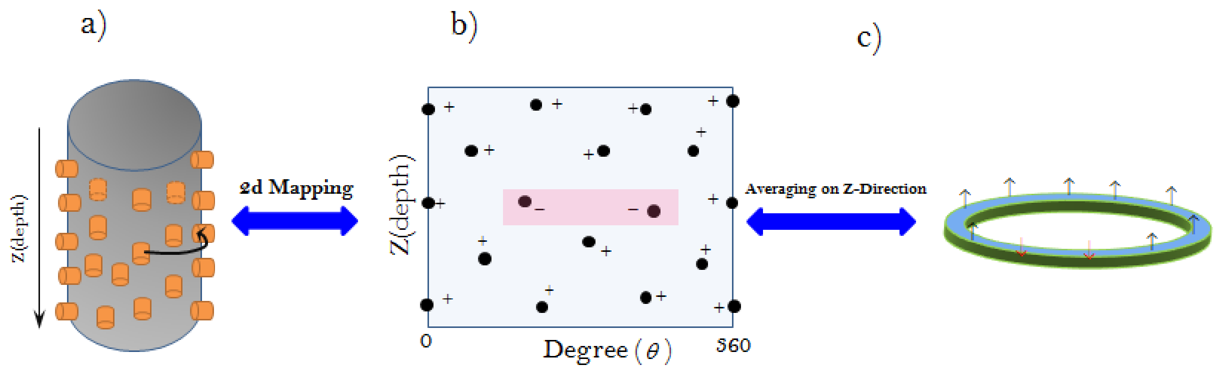

Figure S.12| mapping a multi-array ultrasound sensors set-up around a cylindrical sample on a 2d and 1d structures (ring-like). The +/- signs are schematic representation of K-chain's vectors (states)

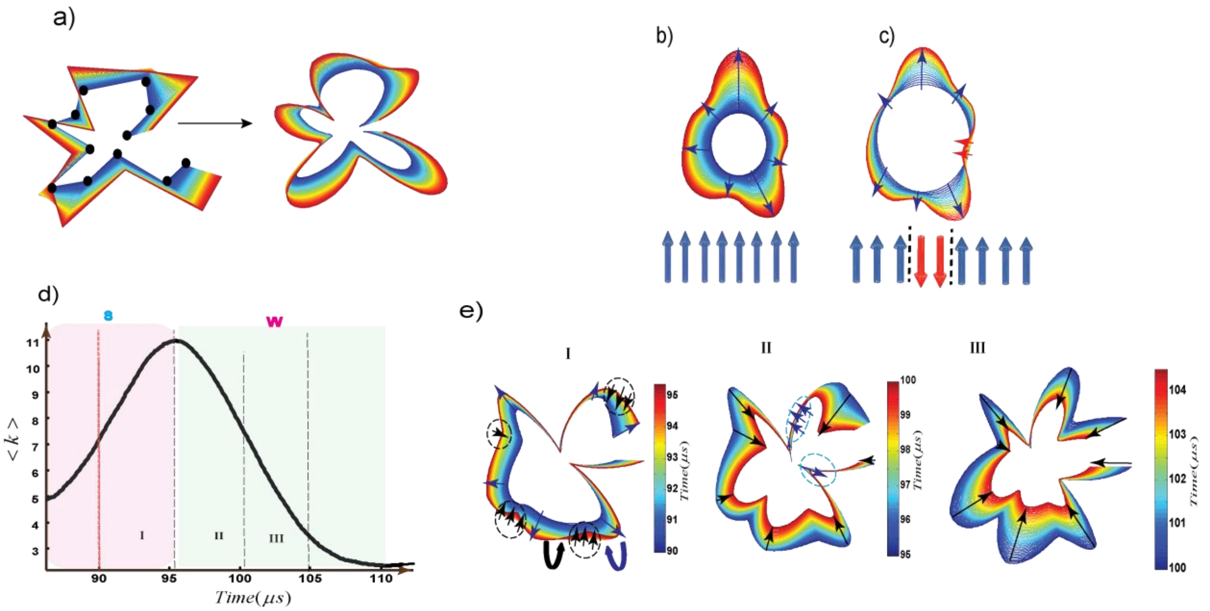

Figure S.13| K-chains mechanism. a) The original K-chain with 12 nodes representing the 12 acoustic sensors mounted around the basalt specimen is smoothed by spline interpolation. The result is a plot of 300 nodes that is easier to visualize. (b,c) K-chains are mapped to spin-like systems where each node is set in analogy with a "spin". (d) An example of the dry event: the average of node degrees (<*k*>) versus time (e) Snapshots of *K*-chains showing the evolution (in 2D polar coordinate system) at three time intervals. Folding (crumpling) of the chain is the source of non-linearity and fragments the network with formation of pairs of kinks/anti-kinks (thick blue and black arrows in the panel "I"). At the third time interval, the system is in a fully polarized (ordered) state. Transition from fully ordered state (e.g., *S*-phase) to another fully ordered state (e.g., W-phase) is associated with the creation of "*kinks*" as the flipped nodes.

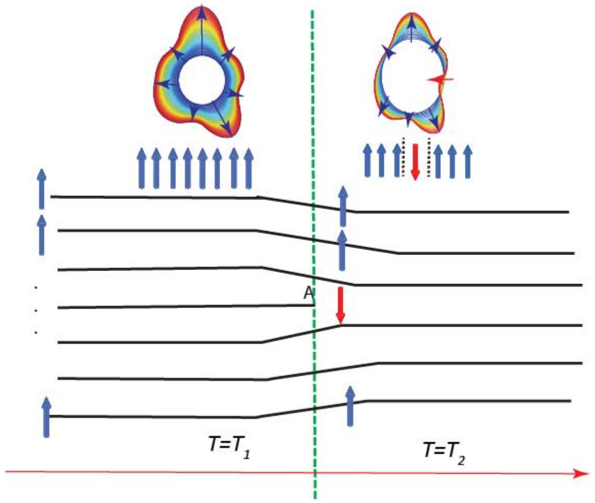

Figure S.14| Spatial patterns of wavefronts (i.e., the surface of constant phase) at two different fixed time steps. An *edge dislocation* occurs at A (modified from *Nye &Berry 1974- also see [8]*). The phase singularity at point A is manifested in a binary network structure as the flipping the node (spin) leading to formation of kink (domain wall) in K-chain.

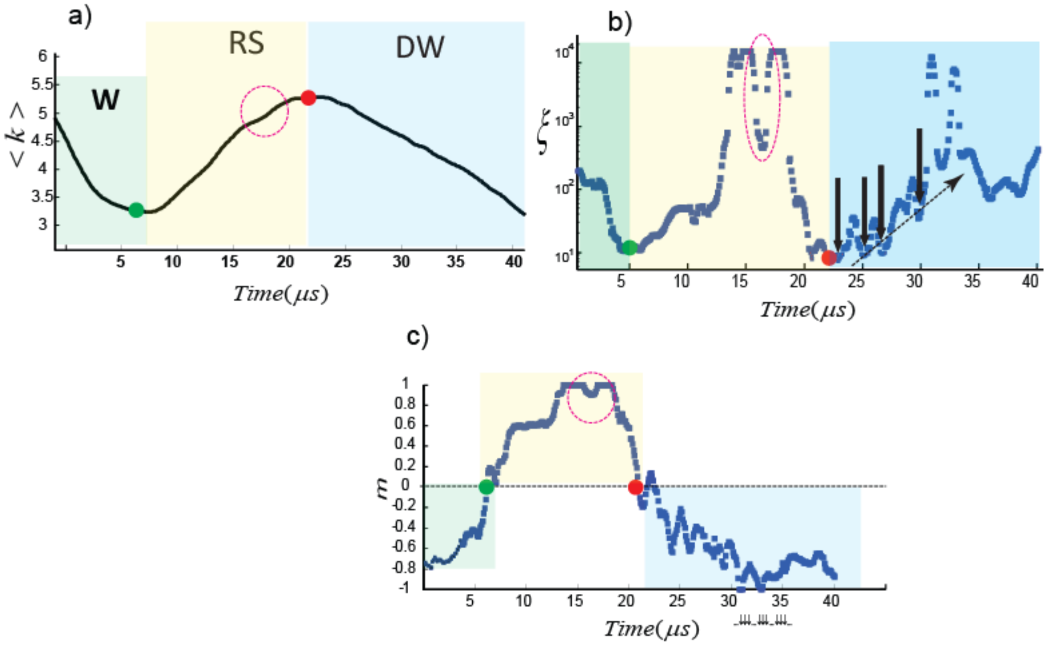

Figure S.15| Structure of the double weakening phase in Wet-cracking noises. (a,b) the mean degree of all nodes for a hybrid event and the corresponding correlation length of *K*-chains . (c) Highly fluctuating order parameter in DW phase drives the system in to the next degenerate state. This signature distinguishes the physics of the secondary weakening phase from the W-phase. We assign this fluctuation to discrete process of squeezing-out of the liquid. The frequency of this almost periodic oscillation is around *200-300kHz*. A double peak in RS phase-exhibited in m(t) and $\xi(t)$ - induces sequence of stiffening-softening-like feature with durations less than 2μs (red dotted-circles).

3-2- W-RS-D transitions: An example of a hybrid event is shown in Figure S.15 (also Fig S.16-17). The nodes with the healing-like characteristics in a way that many nodes become polarized in the same outward direction such that they elongate circumferentially forming large domains in the RS phase, covering a large fraction of the chain's circumference (The dotted red-line around the chain). We have illustrated this characteristic in Fig.S18. Transition from the initial S-phase to fast relaxation regime (W-phase) marks different signatures of kink distribution in comparison with transition from W to RS phase. Approaching fixed points from below or above yields different defect structures. If $k_i < k_{max}$ *(stage 1),* kinks form in a finite size (smaller than the system size-also see [5],[7]). This is the situation for S to W transition and we have described this feature in [5]. In contrast, if $k_i < k_{min}$ *(stage 2)*, a major fraction of the elements form as *string* that spans the whole system and they survive in RS-phase. We have illustrated this feature in Fig.S19 and Fig.4 of the main text. The transition to the RS phase resembles the partial healing of defects (contraction of flipped nodes).

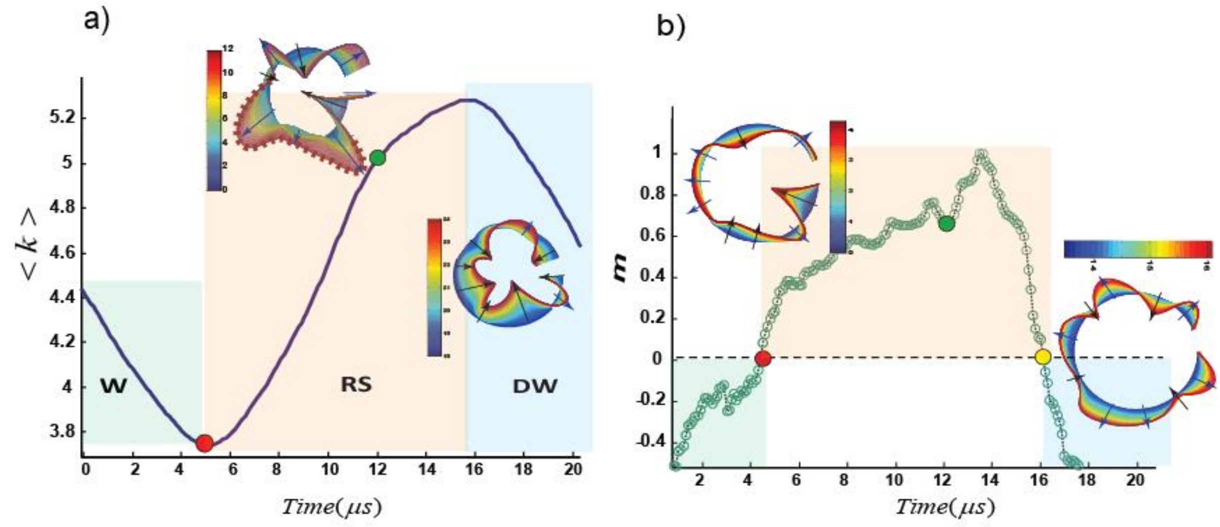

Figure S.16| K-chains patterns and susceptibility in a wet-cracking noise. (a,b) the average number of links versus time and the real-time order parameter. We show the temporal cumulative snapshots of K-chains. The blue and black arrows show expansion and contraction of chains, respectively.
The dotted red-line around the chain in (a) shows the proliferation of healed nodes, resulting in the RS phase. The main defect region in DW phase in panel (a) coincides with the red-dotted healed like line.

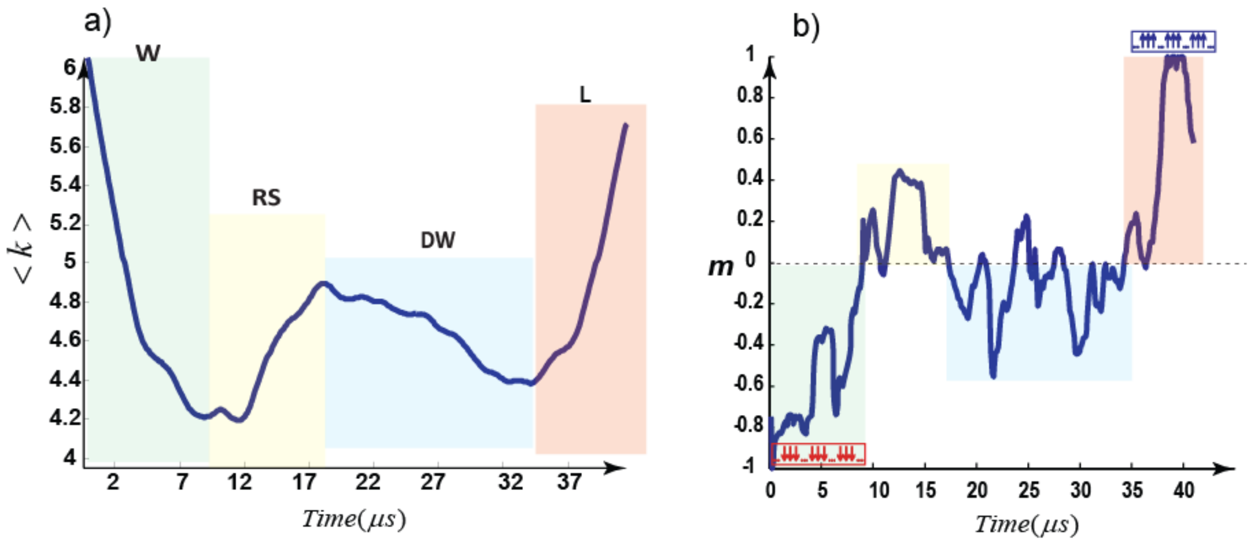

Figure S.17| Another example of a wet-event and W-RS-DW-L transitions. The arrows in (b) indicate the state of K-chains at the beginning and end of the shown time interval. ***m*** **is the order parameter .**

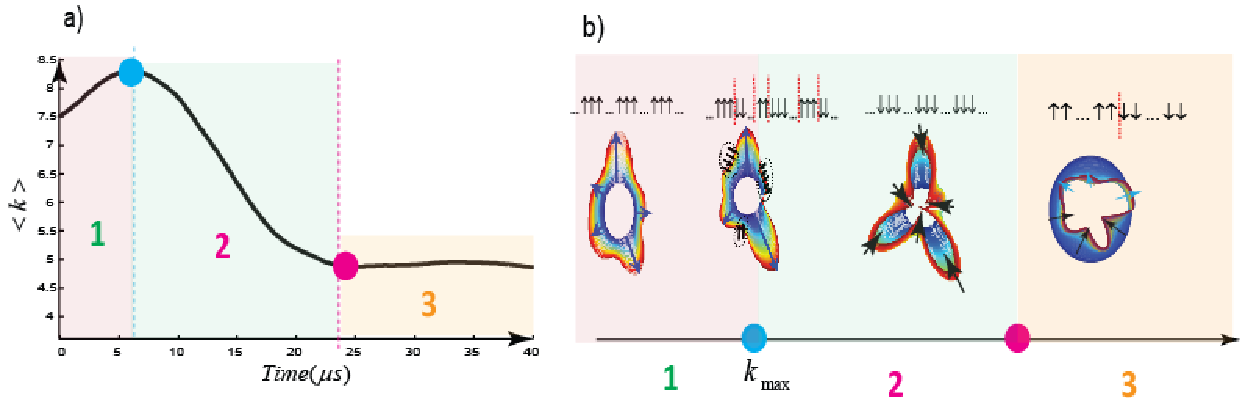

Figure S.18|The crack-like excitation relaxes through at least two fixed points. Approaching fixed points from below or above yields different defects structures. If $k_i \le k_{max\,(stage\ 1)}$, kinks form in a finite size (smaller than the system size-also see [5],[7]). In contrast, if $k_i \le k_{min\,(stage\ 2)}$, a major fraction of the elements form as *string* that spans the system and they survive in RS-phase. Nodes that span the majority of the system disappear at a slower rate leading to stability of RS phase.

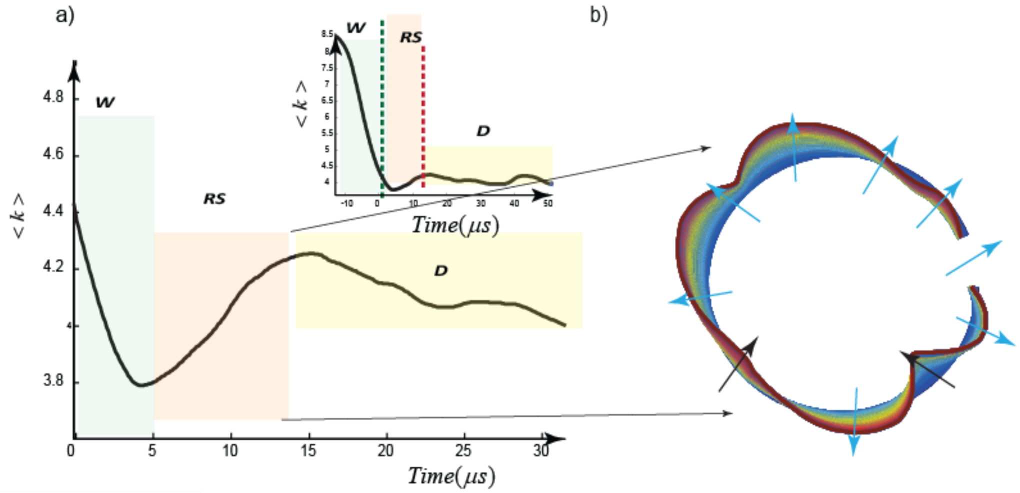

Figure S.19| <u>Dry event</u> : transition to RS-phase is accompanied with the extension of "*healed elements*" (as blue arrow) as a major fraction of the system size form as *string* that spans the system.

3-3-The pair-correlation function (node-node correlation): The discrete equivalent of the autocorrelation function is the two-point correlation function. First we calculate the two-point correlation function (G(x)) in a given time step based on binary-K chains (i.e. fictitious spins). For the calculated functions (for example see [5]), We can fit a correlation function in the form of $G(x) \equiv (1-\frac{x}{L})\exp(-\frac{x}{\xi})$ where L is the total number of the nodes, x is distance, and ξ is the *correlation length*. Correlation length ξ is the cut-off length of the correlation function where for distances shorter than the correlation length, G(x) can be fit by a power law function. For a fully ordered state a triangular function (i.e., fully coherent system and $\xi \to \infty$) is given. In Fig.S20, we show the evolution of W-RS and DW-L transitions and the counterpart correlation length evolution. The transition from the W to RS phase accompanies the fast decline of the correlation length (Fig.S20d-e). The RS-phase is characterized by the extended (proliferated) re-pinning of elements forming a string like structure in K-chains (i.e., outward blue arrows in Fig.S20c) that spans the whole system. As the system approaches the DW phase, the correlation length slowly declines.

A similar pattern of a sudden increase and then slow decline of ξ is observed for the L-phase (Fig.S20e- between 65-80μs). In the context of configurations of K-chains and the observed relaxation phases, we need to suppress slow relaxation phases (D-phase) either intrinsically or extrinsically; the vibrating part of the crack must be confined between the rupture front and repining (i.e., healing) point. For a crack-like excitation the healing occurs when the system approaches thermal equilibrium. To visualize the onset of such re-pining, we might use the states of the nodes in K-chains (Fig.S20). The results shown in Fig.S19-S20 indicates that re-pining process clearly is visualized in K-chain structures as "healed"-like sites in the form of proliferated or extended healed-like elements.

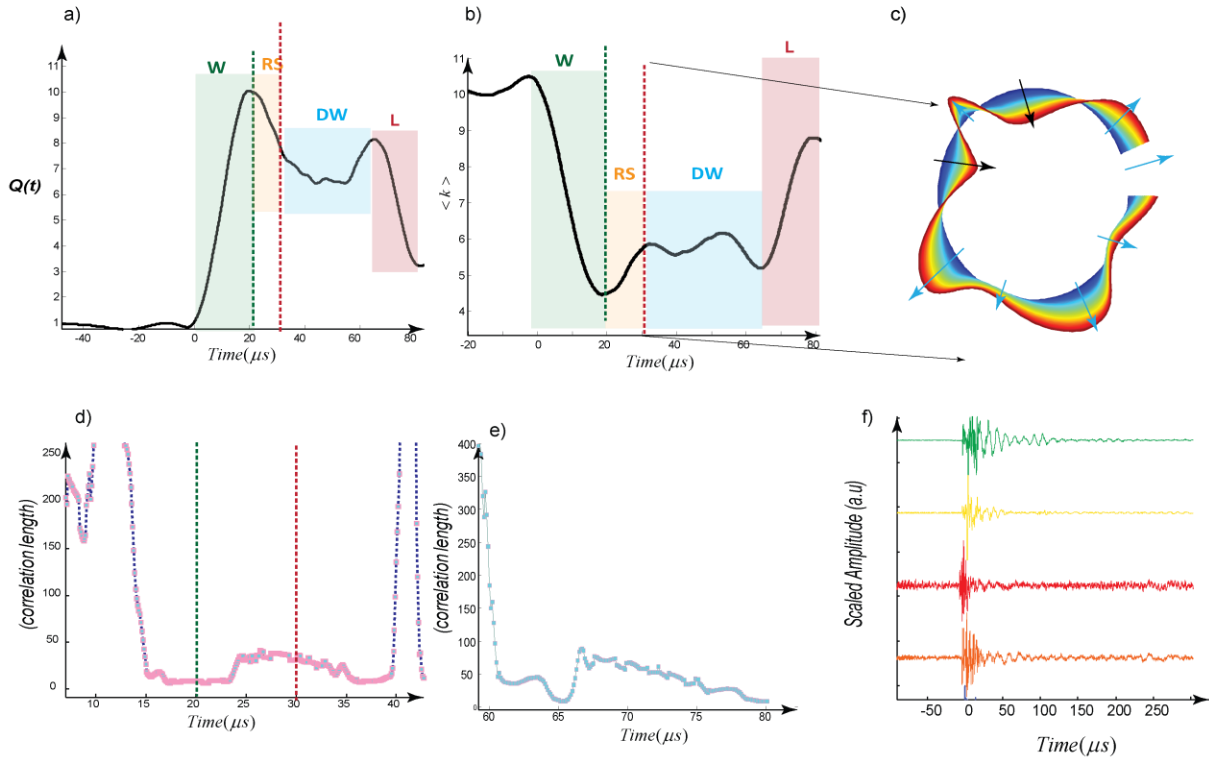

Figure S.20| Correlation function and K-chains of a wet-cracking excitation (a,b) normalized Q(t) and <k(t)> with W-RS-DW-L phases .(c) Accumulated K-chain patterns in RS –phase. The inner (blue) and outer strings (red) are at ~20µs (as the reference point) and ~30µ, respectively. The extended (proliferated) re-pinning elements form a string like structure (outward blue arrows) that spans the major fraction of the system. (d-e) Correlation length (ξ) in W-RS and L phases. Sudden transition from W to RS phase accompanies with almost constant correlation length (frozen correlation length) immediately follows by an enhanced correlation length which decays as the system enters into the DW regime. Note that at~40µs a strong spike appears in DW phase. A similar trend is observed for the L-phase (e). (f) shows the four reordered waveforms for this single event.

References

1- Benson, P. M., Vinciguerra, S., Meredith, P. G., & Young, R. P. Laboratory simulation of volcano seismicity. *Science*, 322(5899), 249-252. (2008).

2- Benson, P. M., Vinciguerra, S., Meredith, P. G., & Young, R. P. Spatio-temporal evolution of volcano seismicity: A laboratory study. *Earth and Planetary Science Letters*, 297(1), 315-323 (2010).

3- P. Benson, A. Schubnel, S. Vinciguerra, C. Trovato, P. Meredith, R.P. Young , Modeling the permeability evolution of micro-cracked rocks from elastic wave velocity inversion at elevated isostatic pressure *J. Geophys. Res.*, 111 (2006), p. B04202 http://dx.doi.org/10.1029/2005JB003710

4- P.M. Benson, B.D. Thompson, P.G. Meredith, S. Vinciguerra, R.P. Young , Imaging slow failure in triaxially deformed Etna basalt using 3D acoustic-emission location and X-ray computed tomography *Geophys. Res. Lett*., 34 (2007), p. L03303 http://dx.doi.org/10.1029/2006GL028721

5- Ghaffari, H. O., W. A. Griffth, P. M. Benson, K. Xia, and R. P. Young. "Observation of the Kibble–Zurek mechanism in microscopic acoustic crackling noises." *Scientific reports* 6 (2016).

6- Ghaffari, H. O., Nasseri, M. H. B. & Young, R. P. Faulting of Rocks in a Three-Dimensional Stress Field by Micro-Anticracks. *Sci. Rep.* 4 (2014).

7- Lin, S.-Z. et al. Topological defects as relics of emergent continuous symmetry and Higgs condensation of disorder in ferroelectrics. *Nature Phys.* 10, 970–977 (2014).

8- Nye, J.F. and Berry, M.V., 1974, January. Dislocations in wave trains. *Proceedings of the Royal Society of London A*: Mathematical, Physical and Engineering Sciences (Vol. 336, No. 1605, pp. 165-190).

9-B.D. Thompson, R.P. Young, D.A. Lockner ,Premonitory acoustic emissions and stick-slip in natural and smooth-faulted Westerly granite *J. Geophys. Res.*, 114 (2009), p. B02205 http://dx.doi.org/10.1029/2008JB005753
